# Pinofuranoxins A and B, Bioactive Trisubstituted Furanones Produced by the Invasive Pathogen *Diplodia sapinea*

**DOI:** 10.1021/acs.jnatprod.1c00365

**Published:** 2021-09-01

**Authors:** Marco Masi, Roberta Di Lecce, Giulia Marsico, Benedetto Teodoro Linaldeddu, Lucia Maddau, Stefano Superchi, Antonio Evidente

**Affiliations:** † Dipartimento di Scienze Chimiche, Università di Napoli Federico II, Complesso Universitario Monte Sant’Angelo, Via Cintia 4, 80126 Napoli, Italy; ‡ Dipartimento di Scienze, Università della Basilicata, Viale dell’Ateneo Lucano 10, 85100 Potenza, Italy; ± Dipartimento Territorio e Sistemi Agro-Forestali, Università di Padova, Viale dell’Università 16, 35020 Legnaro, Italy; § Dipartimento di Agraria, Sezione di Patologia Vegetale ed Entomologia, Università degli Studi di Sassari, Viale Italia 39, 07100 Sassari, Italy

## Abstract

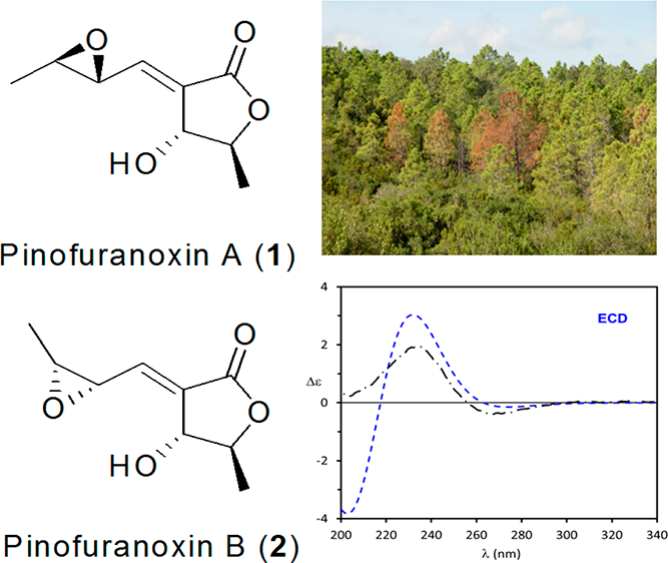

Two new bioactive trisubstituted furanones, named pinofuranoxins A and B (**1** and **2**), were isolated from *Diplodia sapinea*, a worldwide conifer pathogen causing severe disease. Pinofuranoxins A and B were characterized essentially by NMR and HRESIMS spectra, and their relative and absolute configurations were assigned by NOESY experiments and computational analyses of electronic circular dichroism spectra. They induced necrotic lesions on *Hedera helix* L., *Phaseolus vulgaris* L., and *Quercus ilex* L. Compound **1** completely inhibited the growth of *Athelia rolfsii* and *Phytophthora cambivora*, while **2** showed antioomycetes activity against *P. cambivora*. In the *Artemia salina* assay both toxins showed activity inducing larval mortality.

*Diplodia sapinea* (Fr.) Fuckel is one of the most economically important conifer pathogens worldwide. Typical symptoms associated with infection by this pathogen on conifers include tip blight, resinous cankers on the main stem and branches, die-back and a blue stain in the sapwood. The most severe attacks of *D. sapinea* are historically reported in pine plantations affected by environmental stresses such as hail and drought in the southern hemisphere, where its infections are involved in large-scale die-back and tree mortality.^1^ In the northern hemisphere *D. sapinea* has been reported on exotic and native pine species in both Mediterranean and temperate climate areas.^2^ Recent reports in Estonia, Sweden, and Finland^3^ seem to suggest an ongoing geographic range expansion and affirmation of this pathogen in the low-temperature habitats of northern Europe. The same trend of expansion also affects the southern coast of the Mediterranean Sea, where *D. sapinea* has been reported on both conifers and broad-leaved trees in Tunisia and Algeria.^4^ These recent reports highlight the diversity of environmental niches occupied by this invasive species. However, many aspects of the evolutionary success of this pathogen still remain unknown. Overall pathogen fitness includes many components, one of which is the virulence mediated by phytotoxin production.^5^

Despite the ecological impact of *D. sapinea* outbreaks worldwide and the resulting economic losses caused to conifer plantations and timber production, studies on the virulence factors involved in the pathogenesis process and biochemical targets are still limited.^6^ Until now, few phytotoxins are known to be produced by this pathogen. In particular, four nonenolides, named diplodialides A–D, were first isolated and characterized in 1975. Later, two new 5-substituted dihydrofuranones and one 2,4-pyridione, named sapinofuranones A and B and sapinopyridione, were isolated from some *D. sapinea* strains isolated from symptomatic cypress (*Cupressus sempervirens* L. and *Cupressus macrocarpa* Hartw. ex Gordon) in Italy. Finally, three isocumarins, namely, *R*-(−)-mellein, (3*R*,4*R*)-4-hydroxymellein, and (3*R*,4*S*)-4-hydroxymellein, were isolated from a Sardinian strain of *D. sapinea* obtained from *Pinus radiata* D. Don.^6^

Therefore, given the limited information available on the bioactive metabolites produced by *D. sapinea*, a study has been conducted to isolate and characterize the main secondary metabolites produced by a Tunisian strain associated with severe branch canker and die-back of maritime pine (*Pinus pinaster* Aiton).^4^ Two new trisubstituted furanones, named pinofuranoxins A (**1**) and B (**2**), were obtained from the bioguided purification of the organic extract of *D. sapinea*.
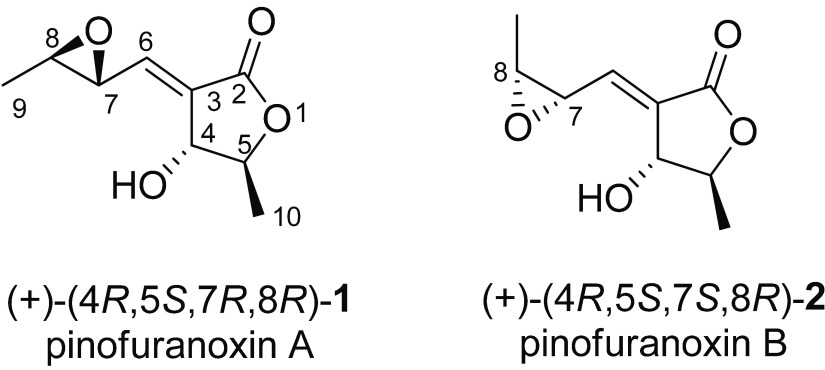


The preliminary ^1^H and ^13^C NMR investigation showed that the two new metabolites **1** and **2** were probably diastereomers, sharing the same molecular formula of C_9_H_12_O_4_. This is consistent with the four indices of hydrogen deficiency, as deduced from their HRESIMS spectra. In addition, their IR and UV spectra showed, in agreement with NMR data, the presence of a conjugated ester carbonyl and a hydroxy group. In particular, the ^13^C and DEPT NMR spectra of pinofuranoxin A (**1**) (Table 1) showed the presence of signals accounting for a carbonyl, five methines, two sp^3^ carbons, of which one was oxygenated, one sp^2^ carbon, and two carbons, probably ascribable to an oxirane ring, a tertiary sp^2^ carbon, and two methyls.

**Table 1:** ^1^H and ^13^C NMR Data of Pinofuranoxins A (**1**) and B (**2**) (^1^H 400 MHz, ^13^C 100 MHz, CDCl_3_)^a^

	**1**			**2**		
no.	δ_C_, type^b^	δ_H_, mult (*J* in Hz)	HMBC^*c*^	δ_C_, type^b^	δ_H_, mult (*J* in Hz)	HMBC^c^
2	169.0, C			168.8, C		
3	133.2, C			135.3, C		
4	72.5, CH	4.68, br s		72.4, CH	4.70, br s	2, 5, 10
5	81.4, CH	4.44, dq (6.7, 3.8)	2, 4, 10	81.5, CH	4.49, dq (6.5, 3.4)	2, 4
6	139.4, CH	6.89, dd (4.1, 2.1)	2, 3, 4, 8	137.0, CH	6.93, dd (3.1, 2.2)	2, 3, 4, 8
7	57.4, CH	3.52, m^d^	3, 6, 8, 9	55.9, CH	3.85, dd (5.5, 3.1)	3, 6, 8
8	57.3, CH	3.06, dq (5.2, 2.1)	9	54.7, CH	3.39, quint (5.5)	6, 7, 9
9	17.6, CH_3_	1.45, d (5.2)	7, 8	13.5, CH_3_	1.34, d (5.5)	7, 8
10	19.9, CH_3_	1.42, d (6.7)	5	20.0, CH_3_	1.42. d (6.5)	4, 5
HO-4		3.52, m^d^	3, 6, 8		3.64, br s	

a COSY and HSQC NMR experiments confirmed the correlations of all the protons and the corresponding carbons.

b Multiplicities were assigned with DEPT.

c HMBC correlations are from proton(s) stated to the indicate carbon.

d These two signals are in part overlapped.

The ^1^H and COSY NMR data (Table 1) showed the presence of an olefinic doublet of doublets at δ 6.89 and a broad singlet of a secondary hydroxylated carbon (H-4) at δ 4.68. H-6 coupled with the multiplet of the proton (H-7) of the adjacent epoxide methine at δ 3.52, which, in turn, coupled with the double quartet of the other adjacent epoxide proton (H-8) at δ 3.06. The latter was also coupled with the geminal methyl (Me-9) at δ 1.45. H-4 allylic coupled (*J* = 2.1 Hz) with H-6^7^ and with the proton (H-5) of the adjacent oxygenated secondary carbon (C-5) at δ 4.44. The latter also coupled with the geminal methyl (Me-10) resonating at δ 1.42. Finally, also the complex multiplet due to a partial overlapping of the hydroxy group at C-4 and the H-7 signal was observed at δ 3.52. The ester carbonyl group, resonating at δ 169.0 in the ^13^C NMR spectrum (Table 1), in the HMBC spectrum (Table 1, Figure 1) coupled with H-5 and H-6, while C-4 and C-5 at δ 72.5 and 81.4 coupled in the same spectrum with H-5 and H-6 and Me-10, respectively.

**Figure 1:**
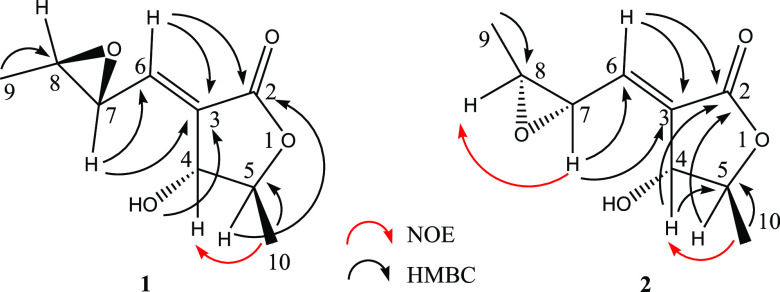
Most significant HMBC (black arrows) and NOE (red arrows) correlations for **1** and **2**.

The 3,4-oxirane-1-pentenyl side chain was located at tertiary sp^2^ C-3 by its couplings in the HMBC spectrum (Table 1, Figure 1) with H-6 and HO-4. The chemical shifts for all of the protons and the corresponding carbons were assigned and are listed in Table 1. These are consistent with those previously reported for other some trisubstituted γ-lactones.^8^ Thus, **1** was formulated as 4-hydroxy-5-methyl-3-((3-methyloxiran-2-yl)methylene)dihydrofuran-2(3*H*)-one.

Pinofuranoxin B (**2**), as cited above, showed the same molecular formula and structural features as **1**, suggesting their diastereomeric relationship. In particular, their NMR and HRESIMS spectra were very similar. All of the ^1^H and ^13^C NMR data of **2** listed in Table 1 were obtained from its 1D and 2D NMR spectra (COSY, HSQC, and HMBC). However, a significant difference was observed in the NOESY spectra (Figure 1, Table S1).

In both **1** and **2** the correlations between H-4 and Me-10 and the lack of one between H-4 and H-5 supported a *trans*-substitution of the dihydrofuranone ring. The correlation observed only in **2** between H-7 and H-8 allowed a *cis*- and a *trans*-substitution of the oxirane ring in **2** and **1** to be assigned, respectively. The configuration of the double bond was deduced from the absence of coupling between H-6 and H-4 in the NOESY spectra of both **1** and **2**. In addition the chemical shifts of H-6 and C-6 were very similar to those of protons and carbons of some natural furanones bearing an α *E*-disubstituted vinyl group, which significantly differed from those having a *Z*-vinyl group.^8,9^ Thus, **2** was formulated as a diastereomer of **1** by inversion of the configuration on the oxirane ring. The two metabolites are characterized by a unique combination of functional groups among all the classes of naturally occurring compounds. In fact, although some 2-alkylidene-3-hydroxy-4-methyl-butanolides are known to be bioactive metabolites of *Lauraceae* plants,^9^ only a single compound of this family has been obtained from a fungus of marine sponges.^10^ Moreover, this is the first example in which the presence of an epoxy moiety is reported in such structures.

Once the structures of (+)-**1** and (+)-**2** had been determined, the absolute configuration (AC) of both compounds were assigned by computational analysis of their electronic circular dichroism (ECD) spectra,^11^ an approach that has proven to be particularly reliable and straightforward for the AC assignment in solution of complex chiral compounds^12^ including natural products.^13,14^ Accordingly, the ECD spectra of **1** and **2** were recorded in MeCN in the 200–340 nm range. The ECD spectrum of (+)-**1** (Figure 2) displays a weak broad negative Cotton effect (CE) centered at about 263 nm (Δε −0.39) followed by a more intense positive one at 234 nm (Δε +1.93), while that of (+)-**2** (Figure 3) shows two oppositely signed CEs: a weaker negative one at 270 nm (Δε −0.34) and a more intense positive band at 231 nm (Δε +4.36).

**Figure 2:**
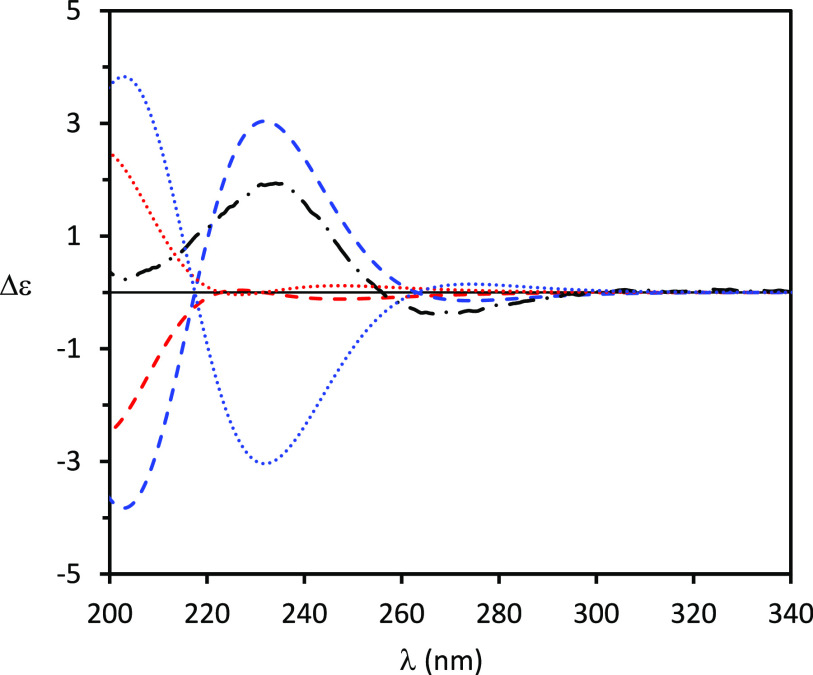
Comparison between experimental ECD spectra (dashed-dotted black line) of (+)-**1** with calculated [TDDFT/CAM-B3LYP/aug-cc-pVDZ/IEFPCM(MeCN)] ones. Computed ECD spectrum for (4*S*,5*R*,7*R*,8*R*)-**1a** (dotted red line), (4*R*,5*S*,7*S*,8*S*)-*ent***-1a** (dashed red line), (4*S*,5*R*,7*S*,8*S*)-**1b** (dotted blue line), and (4*R*,5*S*,7*R*,8*R*)-*ent***-1b** (dashed blue line). The calculated ECD spectra have been divided by 2. Conformers with intramolecular hydrogen bonding have been discarded (see text).

**Figure 3:**
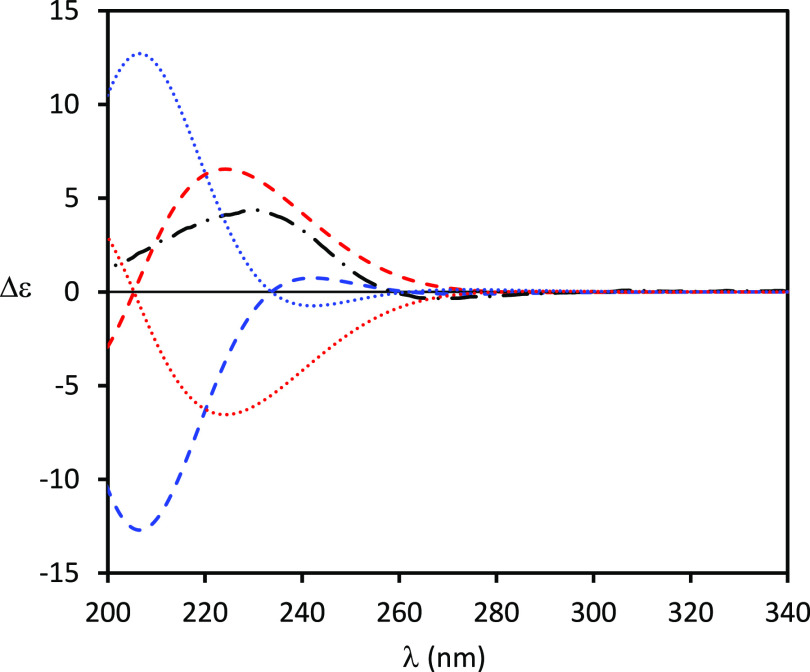
Comparison between experimental ECD spectra (dashed-dotted black line) of (+)-**2** with calculated [TDDFT/CAM-B3LYP/aug-cc-pVDZ/IEFPCM(MeCN)] ones. Computed ECD spectrum for (4*S*,5*R*,7*R*,8*S*)-**2a** (dotted red line), (4*R*,5*S*,7*S*,8*R*)-*ent***-2a** (dashed red line), (4*S*,5*R*,7*S*,8*R*)-**2b** (dotted blue line), and (4*R*,5*S*,7*R*,8*S*)-*ent***-2b** (dashed blue line). Conformers with intramolecular hydrogen bonding have been discarded (see text).

A computational analysis of these chiroptical data by DFT was then undertaken. When both relative and AC are unknown, like in the case of **1** and **2**, chiroptical data for any possible stereoisomer should be computed and compared with the experimental data.^15^ However, because the NOESY NMR studies provided information about the relative configurations of some stereocenters, it was not necessary to take into account all the possible 2^4^ stereoisomers of **1** and **2**. In particular, NOESY allows the *trans* (4S*,5R*) relative configuration to be assigned to the two stereocenters of the lactone ring in both **1** and **2**. Moreover, compound **1**, displaying a *trans*-configuration at the oxirane ring, has either a (7*R*,8*R*) or (7*S*,8*S*) AC of the two C-7 and C-8 stereocenters, while in compound **2**, having a *cis* oxirane ring, these two stereocenters must have either a (7*R*,8*S*) or a (7*S*,8*R*) AC. It follows that the computational analysis can be performed on the (4S*,5R*,7R*,8R*) and (4S*,5R*,7S*,8S*) diastereomers for the *trans* compound **1** and on the (4S*,5R*,7R*,8S*) and (4S*,5R*,7S*,8R*) diastereomers for the *cis* compound **2**. Since enantiomeric diastereomers have mirror-image ECD spectra, for each compound it is sufficient to predict the chiroptical properties for only one enantiomer of the possible diastereomers. In the case of **1**, conformational analysis on the arbitrarily chosen stereoisomers (4*S*,5*R*,7*R*,8*R*)**-1a** and (4*S*,5*R*,7*S*,8*S*)**-1b** provided four appreciably populated conformers for both (Tables S2 and S3; Figures S1 and S2; Supporting Information). The two most abundant conformers of **1a** and **1b** accounting for about 70% and 46% of the overall population, respectively, display the methyl and hydroxy substituents of the lactone ring in equatorial arrangement. Investigation of the conformer ensemble revealed that in the second most populated conformer of **1a** and in the third most populated conformer of **1b** hydrogen bonding occurs between the hydroxy moiety and oxirane ring. Taking into account that the polarized continuum solvation model (PCM) employed in these computations accounts for bulk solvent effects, but is unsuited to describe the ability of the solvent to directly participate as a hydrogen-bond acceptor, we considered the intramolecular H-bonded conformers provided by PCM computations in MeCN as computational artifacts.^16^ Therefore, we decided to discard those conformers displaying an intramolecular H-bonding in the Boltzmann averaging of the computed ECD spectra. The ECD spectra for both **1a** and **1b** diastereomers were then calculated for each chosen conformer and Boltzmann averaged over the conformers’ populations.

Comparison of the ECD experimental spectrum with the computed ones of **1a** and **1b** and the mirror-image spectra of their enantiomers (4*R*,5*S*,7*S*,8*S*)**-***ent***-1a** and (4*R*,5*S*,7*R*,8*R*)**-***ent***-1b** (Figure 2) shows a quite good agreement between the experimental and the spectrum of the *ent***-1b** stereoisomer, while those of the stereoisomers *ent***-1a**, **1a**, and **1b** can be safely ruled out. This result then allows the (4*R*,5*S*,7*R*,8*R*) AC to be reliably assigned to (+)-pinofuranoxin A ((+)-**1**). Notably, computed ECD spectra of **1a** and **1b** diastereomers, obtained taking into account all the possible conformers, including H-bonded ones, appear instead quite similar and do not allow a reliable AC assignment (Figure S5). Further confirmation of this AC assignment was also obtained by simulating the solvent effects by an explicit approach (Supporting Information). The same analysis was performed for (+)-pinofuranoxin B ((+)-**2**). Computational conformational analysis on the arbitrarily chosen diastereoisomers (4*S*,5*R*,7*R*,8*S*)-**2a** and (4*S*,5*R*,7*S*,8*R*)-**2b** provided four and five populated conformers for **2a** and **2b**, respectively (Tables S4 and S5; Figure S3 and Figure S4). For the same consideration as above, we discarded conformers displaying an intramolecular H-bond and Boltzmann averaged ECD spectra of the remaining ones. Comparison of the ECD experimental spectra with the computed ones for **2a** and **2b** and of their enantiomers (4*R*,5*S*,7*S*,8*R*)-*ent***-2a** and (4*R*,5*S*,7*R*,8*S*)-*ent***-2b** (Figure 3) shows the quite good agreement between the experimental spectrum and that of the *ent***-2a** stereoisomer, while the spectra of *ent***-2b**, **2a**, and **2b** are in greater disagreement, allowing these stereoisomers to be ruled out. Also in this case, computed ECD spectra obtained taking into account all the possible conformers would not allow a reliable AC assignment (Figure S6), while computations employing the explicit solvent approach confirmed the above assignment (Supporting Information), reliably establishing the (4*R*,5*S*,7*S*,8*R*) AC for (+)-pinofuranoxin B ((+)-**2**).

Pinofuranoxins A (**1**) and B (**2**) were then screened for phytotoxic, antifungal, antioomycetes, and zootoxic activities. Pinofuranoxins A (**1**) and B (**2**) at a concentration of 1 mg/mL caused necrotic lesions on all the plants tested, with area lesion sizes of 112, 46, and 61 mm^2^ and 79, 55, and 50 mm^2^, respectively, on English ivy, bean, and holm oak leaves. Compound **1** induced necrotic effects also at 0.5 and 0.1 mg/mL, while **2** has no phytotoxic effects at 0.1 mg/mL on all plant species (Table 2).

**Table 2:** Phytotoxicity Data for Pinofuranoxins A (**1**) and B (**2**)

		leaf puncture bioassay^a^		
compound	concentration (mg/mL)	English ivy	bean	holm oak
**1**	1.0	112 ± 14	46 ± 5	61 ± 10.
	0.5	49 ± 5	40 ± 2	36 ± 7
	0.1	5 ± 1	7 ± 1	1 ± 0
**2**	1.0	79 ± 3	55 ± 10	50 ± 2
	0.5	14 ± 3	27 ± 3	23 ± 2
	0.1	na	na	na

a Data are expressed as median area lesion ± error standard (mm^2^); na = inactive.

Compounds **1** and **2** were also tested against two plant pathogenic fungi (*Athelia rolfsii* and *Diplodia corticola*) and the oomycota *Phytophthora cambivora*. Pentachloronitrobenzene (PCNB) and metalaxyl-M were used as positive controls depending on the species. Compound **1** at a concentration of 0.2 and 0.1 mg/plug completely inhibited the mycelial growth of *P. cambivora* and *A. rolfsii*, while *D. corticola* seems to be more resistant. Compound **2** completely inhibited *P. cambivora* at both concentrations, whereas it did not show antifungal activity against *A. rolfsii* and *D. corticola* (Table 3).

**Table 3:** Inhibitory Activity of Pinofuranoxins A (**1**) and B (**2**) against Agrarian and Forest Phytopathogens

		mycelial growth inhibition (%)		
compound	concentration (mg/plug)	*Athelia rolfsii*	*Diplodia corticola*	*Phytophthora cambivora*
**1**	0.2	100	38	100
	0.1	100	21	100
**2**	0.2	na^a^	na	100
	0.1	na	na	100
PCNB	0.2	81	72	nt^b^
	0.1	76	69	nt
metalaxyl-M	0.2	nt	nt	100
	0.1	nt	nt	100

a na = inactive.

b nt = not tested.

Compounds **1** and **2** caused 96% and 51% of larval mortality in a brine shrimp (*Artemia salina* L.) assay^17^ at 200 μg/mL, whereas at the other two concentrations (100 and 50 μg/mL) larval mortality was less than 20% for **1**, while **2** was inactive.

Although dihydrofuranones and epoxide derivatives are well known as natural occurring compounds^18^ and also as fungal phytotoxins,^19^ pinofuronoxins A (**1**) and B (**2**) have a unique combination of functional groups. They showed similar phytotoxic activity but a different antifungal and zootoxic activity. Both the α,β-unsaturated carbonyl (involved in nucleophilic Michael addition) and epoxide ring (involved in nucleophilic substitution), present in the two toxins, are well-known structural features frequently reported to impart biological activities.^19^ The different antifungal and zootoxic activity could be ascribed to the different absolute and relative configuration of the epoxy ring.

As currently there are relatively few fungicides to control *Phytophthora*-related diseases, the antimicrobial tests revealed a potential application for pinofuranoxin B.^20^ These findings emphasize the potential of *Diplodia* species for the discovery of new natural bioactive compounds with possible applications in agriculture and medicine.^21^

## Experimental Section

### General Experimental Procedures

Optical rotations were measured in a MeOH solution on a Jasco P-1010 digital polarimeter; UV spectra were recorded on a JASCO V-530 spectrophotometer in CH_3_CN solution, while ECD spectra were recorded at room temperature on a JASCO J815 spectropolarimeter, by using 0.1 mm cells; IR spectra were recorded as a glassy film on a PerkinElmer Spectrum One Fourier transform infrared spectrometer. ^1^H and ^13^C NMR spectra were recorded at 400 and 100 MHz, respectively, in CDCl_3_ on a Bruker spectrometer. The same solvent was used as a specific chemical shift reference of 7.26 and 77.0 ppm, respectively. Carbon multiplicities were determined by DEPT spectra. DEPT, COSY-45, HSQC, HMBC, and NOESY experiments were performed using Bruker. HRESI and ESI mass spectra were performed as previously described.^22^ Analytical and preparative thin-layer chromatography (TLC) were performed on silica gel plates (Kieselgel 60, F_254_, 0.25 and 0.5 mm, respectively) or on reverse-phase (Whatman, KC18 F_254_, 0.20 mm) plates. The compounds were visualized by exposure to UV light and/or iodine vapors and/or by spraying first with 10% H_2_SO_4_ in MeOH and then with 5% phosphomolybdic acid in EtOH, followed by heating at 110 °C for 10 min. Column chromatography (CC) was carried out on silica gel (Merck, Kieselgel 60, 0.063–0.200 mm).

### Fungal Strain

The *D. sapinea* strain used in this study was originally isolated from a cankered branch of maritime pine collected in a declining stand located in northwest Tunisia.^4^ The strain was identified on the basis of morphological characters and analysis of internal transcribed spacer (ITS) rDNA. Fungal DNA extraction, PCR amplification reactions, and DNA sequencing were conducted as reported by Linaldeddu et al. (2016).^23^ The sequence of the ITS region has been deposited in GenBank (accession number: MW436711). Pure cultures were maintained on potato-dextrose agar (PDA 39 g/L, Oxoid Ltd., Basingstoke, UK) and stored at 4 °C in the collection of the Dipartimento di Agraria, University of Sassari, Italy, as C3.

### Production, Extraction, and Purification of the Metabolites

The fungus was grown on liquid medium (Czapek amended with 2% corn meal; pH 5.7). The culture filtrates (5 L) were extracted exhaustively with EtOAc, yielding an oily brown residue (316 mg). The latter was bioguided purified by CC, eluting with CHCl_3_/iPrOH (85:15, v/v), and 10 homogeneous fractions were collected. The residue (10.9 mg) of the fourth fraction was purified by TLC, eluting with *n*-hexane/EtOAc (1:1, v/v), yielding an oily homogeneous compound named pinofuranoxin A (**1**, 3.4 mg, *R*_*f*_ of 0.27). The residue (77.1 mg) of the fifth fraction was purified by TLC eluting with *n*-hexane/CHCl_3_/iPrOH (7:2:1, v/v/v), affording four fractions. The residue of the third fraction (33.5 mg) was purified by TLC, eluting with CHCl_3_/iPrOH (98:2, v/v), yielding five fractions. The residue of the first fraction was an oily homogeneous compound and was named pinofuranoxin B (**2**, 4.5 mg, *R*_*f*_ of 0.14). The residue of the second fraction of the latter purification was further purified by TLC, eluting with *n*-hexane/EtOAc (1:1, v/v), yielding a further amount of pinofuranoxin A (**1**, 12.5 for a total of 15.9 mg).

#### Pinofuranoxin A (**1**):

amorphous solid, [α]^25^_D_ +22.4 (*c* 0.34, MeOH); UV (CH_3_CN) λ_max_ (log ε) 227 (4.1) nm; ECD (4.54 × 10^–3^ M, MeCN) λ_max_ (Δε) 234 (+1.93), 263 (−0.39) nm; IR ν_max_ 3393, 1725, 1654, 1266 cm^–1^; ^1^H and ^13^C NMR, Table 1; HRESIMS *m*/*z* 407.1111 [2 M + K]^+^ (calcd for C_18_H_24_KO_8_, 407.1108), 391.1373 [2 M + Na]^+^ (calcd for C_18_H_24_NaO_8_, 391.1369), 373.1259 [2 M + Na – H_2_O]^+^ (calcd for C_18_H_22_NaO_7_, 373.1263), 185.0823 [M + H]^+^ (calcd for C_9_H_13_O_4_, 185.0823), 167.0707 [M + H – H_2_O]^+^ (calcd for C_9_H_11_O_3_, 167.0708).

#### Pinofuranoxin B (**2**):

amorphous solid, [α]^25^_D_ +93.1 (*c* 0.45 MeOH); UV (CH_3_CN) λ_max_ nm (log ε) 231 (4.3); ECD (4.37 × 10^–3^ M, MeCN) λ_max_ (Δε) 231 (+4.36), 270 (−0.34) nm; IR ν_max_ 3393, 1745, 1654, 1296 cm^–1^; ^1^H and ^13^C NMR see Table 1; HRESIMS *m*/*z* 407.1114 [2 M + K]^+^ (calcd for C_18_H_24_KO_8_, 407.1108), 391.1369 [2 M + Na]^+^ (calcd for C_18_H_24_NaO_8_, 391.1369), 373.1260 [2 M + Na – H_2_O]^+^ (calcd for C_18_H_22_NaO_7_, 373.1263), 185.0807 [M + H]^+^ (calcd for C_9_H_13_O_4_, 185.0823), 167.0712 [M + H – H_2_O]^+^ (calcd for C_9_H_11_O_3_, 167.0708).

### Leaf Puncture Assay

English ivy (*Hedera helix* L.), bean (*Phaseolus vulgaris* L.), and holm oak (*Quercus ilex* L.) leaves were used for this assay. Each compound was tested at 1.0, 0.5, and 0.1 mg/mL. The assay was performed as previously reported by Andolfi et al.^24^ Each treatment was repeated three times. Leaves were observed daily and scored for symptoms after 5 days. The effect of the toxins on the leaves was observed up to 10 days. Lesions were estimated using APS Assess 2.0 software following the tutorials in the user’s manual.^25^ The lesion size was expressed in mm^2^.

### Antifungal Assays

Compounds **1** and **2** were preliminarily tested on three plant pathogens, *Athelia rolfsii* (Curzi) C.C. Tu & Kimbr., *Diplodia corticola* A.J.L. Phillips, A. Alves & J. Luque, and *Phytophthora cambivora* (Petri) Buisman. The sensitivity of the three pathogens to two compounds was evaluated, on carrot agar (CA) (*P. cambivora*) or PDA (*A. rolfsii* and *D. corticola*), as inhibitors of the mycelial radial growth. The assay was performed as previously reported by Masi et al.^26^ Each compound was tested at 200 and 100 μg/plug. MeOH was used as negative control. Metalaxyl-M (Mefenoxam; p.a. 43.88%; Syngenta), a synthetic fungicide to which the oomycetes are sensitive, and PCNB for ascomycetes and basidiomycetes were used as positive controls. Each treatment consisted of three replicates, and the experiment was repeated twice.

### *Artemia salina* Bioassay

The *in vitro* toxic effects of **1** and **2** were also evaluated on brine shrimp larvae (*Artemia salina* L.). The assay was performed in cell culture plates with 24 cells (Corning) as previously reported by Andolfi et al. (2014).^24^ The metabolites were tested at 200, 100, and 50 μg/mL. Tests were performed in quadruplicate. The percentage of larval mortality was determined after 36 h of incubation at 27 °C in the dark.

## Supporting Information Available

1D and 2D ^1^H and ^13^C NMR and HRESIMS spectra of **1** and **2**; computational details; experimental and computed UV and ECD spectra; structures and populations of computed conformers (PDF)
