# Supplementary material for: Pinofuranoxins A and B, Bioactive Trisubstituted Furanones Produced by the Invasive Pathogen Diplodia sapinea
Source: J Nat Prod. 2021 Sep 1;84(9):2600–5. doi: 10.1021/acs.jnatprod.1c00365 (PMC8477388; doi:10.1021/acs.jnatprod.1c00365)
Supplement: Supplementary file 1 — np1c00365_si_001.pdf [file np1c00365_si_001.pdf]

# SUPPORTING INFORMATION

# Pinofuranoxins A and B, Bioactive Tetrasubstituted Furanones Produced by the Invasive Pathogen *Diplodia sapinea*

Marco Masi,† Roberta Di Lecce,† Giulia Marsico,‡ Benedetto Teodoro Lunaldeddu,± Lucia Maddau,§ Stefano Superchi,‡ and Antonio Evidente*,†

†Dipartimento di Scienze Chimiche, Università di Napoli Federico II, Complesso Universitario Monte Sant'Angelo, Via Cintia 4, 80126, Napoli, Italy

‡Dipartimento di Scienze, Università della Basilicata, Viale dell'Ateneo Lucano 10, 85100 Potenza, Italy.

±Dipartimento Territorio e Sistemi Agro-Forestali, Università di Padova, Viale dell'Università 16, Legnaro 35020, Italy

§Dipartimento di Agraria, Sezione di Patologia Vegetale ed Entomologia, Università degli Studi di Sassari, Viale Italia 39, 07100, Sassari, Italy

## Supporting Information List

Page 3: **Table S1.** NOESY data of pinofuronoxins A and B (**1** and **2**)

Page 4: **Table S2.** Conformers Boltzmann distribution of (4*S*,5*R*,7*R*,8*R*)-**1a**.

Page 4: **Figure S1.** Minimum energy conformers of (4*S*,5*R*,7*R*,8*R*)-**1a**.

Page 5: **Table S3.** Conformers Boltzmann distribution of (4*S*,5*R*,7*S*,8*S*)-**1b**.

Page 5: **Figure S2.** Minimum energy conformers of (4*S*,5*R*,7*S*,8*S*)-**1b**.

Page 6: **Table S4.** Conformers Boltzmann distribution of (4*S*,5*R*,7*R*,8*S*)-**2a**.

Page 6: **Figure S3.** Minimum energy conformers of (4*S*,5*R*,7*R*,8*S*)-**2a**.

Page 7: **Table S5.** Conformers Boltzmann distribution of (4*S*,5*R*,7*S*,8*R*)-**2b**.

Page 7: **Figure S4.** Minimum energy conformers of (4*S*,5*R*,7*S*,8*R*)-**2b**.

Page 8: **Figure S5.** Experimental and calculated UV and ECD spectra for diastereoisomers of **1**.

Page 8: **Figure S6.** Experimental and calculated ECD spectra for diastereoisomers of **2**.

Page 9: ECD computations with explicit solvent model.

Page 10: **Table S6.** Conformers Boltzmann distribution of (4*S*,5*R*,7*R*,8*R*)-**1a·ACN** adduct.

Page 10: **Figure S7.** Minimum energy conformers of (4*S*,5*R*,7*R*,8*R*)-**1a·ACN** adduct.

Page 11: **Table S7.** Conformers Boltzmann distribution of (4*S*,5*R*,7*S*,8*S*)-**1b·ACN** adduct.

Page 11: **Figure S8.** Minimum energy conformers of (4*S*,5*R*,7*S*,8*S*)-**1b·ACN** adduct.

Page 12: **Figure S9.** Experimental UV and ECD spectra of **1** and calculated UV and ECD spectra for diastereoisomers of **1·ACN** adduct.

Page 13: **Table S8.** Conformers Boltzmann distribution of (4*S*,5*R*,7*R*,8*S*)-**2a·ACN** adduct.

Page 13: **Figure S10.** Minimum energy conformers of (4*S*,5*R*,7*R*,8*S*)-**2a·ACN** adduct.

Page 14: **Table S9.** Conformers Boltzmann distribution of (4*S*,5*R*,7*S*,8*R*)-**2b·ACN** adduct.

Page 14: **Figure S11.** Minimum energy conformers of (4*S*,5*R*,7*S*,8*R*)-**2b·ACN** adduct.

Page 15: **Figure S12.** Experimental UV and ECD spectra of **2** and calculated UV and ECD spectra for diastereoisomers of **2·ACN** adduct.

Page 16: **Spectrum 1**. $^1$H NMR spectrum of pinofuranoxin A (**1**) ($CDCl_3$, 400 MHz).

Page 16: **Spectrum 2**. $^{13}$C NMR spectrum of pinofuranoxin A (**1**) ($CDCl_3$, 100 MHz).

Page 17: **Spectrum 3.** DEPT-135 NMR spectrum of pinofuranoxin A (**1**) ($CDCl_3$, 100 MHz).

Page 17: **Spectrum 4**. HSQC spectrum of pinofuranoxin A (**1**) ($CDCl_3$, 400/100 MHz).

Page 18: **Spectrum 5**. HMBC spectrum of pinofuranoxin A (**1**) ($CDCl_3$, 400/100 MHz).

Page 18: **Spectrum 6**. COSY spectrum of pinofuranoxin A (**1**) ($CDCl_3$, 400 MHz).

Page 19: **Spectrum 7**. NOESY spectrum of pinofuranoxin A (**1**) ($CDCl_3$, 400 MHz).

Page 19: **Spectrum 8.** HR ESIMS spectrum of pinofuranoxin A (**1**).

Page 20: **Spectrum 9**. $^1$H NMR spectrum of pinofuranoxin B (**2**) ($CDCl_3$, 400 MHz).

Page 20: **Spectrum 10**. $^{13}C$ NMR spectrum of pinofuranoxin B (**2**) ($CDCl_3$, 100 MHz).

Page 21: **Spectrum 11.** DEPT-135 NMR spectrum of pinofuranoxin B (**2**) ($CDCl_3$, 100 MHz).

Page 21: **Spectrum 10**. HSQC spectrum of pinofuranoxin B (**2**) ($CDCl_3$, 400/100 MHz).

Page 22: **Spectrum 11**. HMBC spectrum of pinofuranoxin B (**2**) ($CDCl_3$, 400/100 MHz).

Page 22: **Spectrum 12**. COSY spectrum of pinofuranoxin B (**2**) ($CDCl_3$, 400 MHz).

Page 23: **Spectrum 13**. NOESY spectrum of pinofuranoxin B (**2**) ($CDCl_3$, 400 MHz).

Page 23: **Spectrum 14.** HR ESIMS spectrum of pinofuranoxin B (**2**).

**Table S1. NOESY Data of Pinofuranoxins A and B (1 and 2)**

| **1** | | **2** | |
|---|---|---|---|
| Irradiated | Observed | Irradiated | Observed |
| H-4 | Me-10 | H-4 | Me-10 |
| H-5 | Me-10 | H-5 | Me-10 |
| H-8 | Me-9 | H-7 | H-8 |
| | | H-8 | Me-9 |

**Table S2.** Conformers Boltzmann distribution of (4*S*,5*R*,7*R*,8*R*)-**1a**.

| DFT/B3LYP/6-311++G(2d,2p)/(IEFPCM($CH_3CN$) | | |
|---|---|---|
| **Conformers** | **ΔG (Kcal/mol)** | **% Pop** |
| **C1** | 0.000000 | 39.3 |
| **C2** | 0.212553 | 27.4 |
| **C3** | 0.491568 | 17.1 |
| **C4** | 0.526053 | 16.2 |

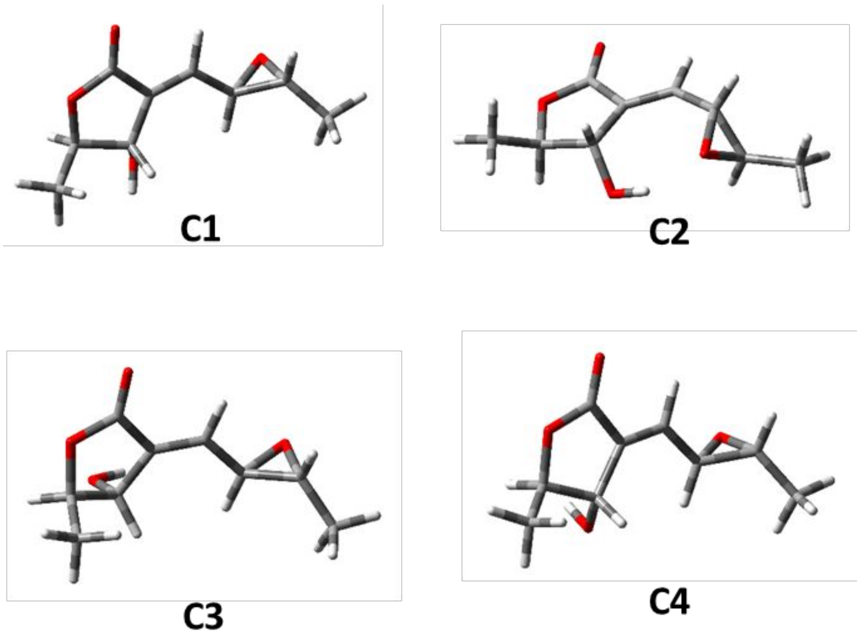


**Figure S1.** Minimum energy conformers of (4*S*,5*R*,7*R*,8*R*)-**1a** computed at DFT/B3LYP/6-311++G(2d,2p)/IEFPCM($CH_3CN$) level. Conformers C2 displays intramolecular H-bond.

**Table S3.** Conformers Boltzmann distribution of (4*S*,5*R*,7*S*,8*S*)-**1b**.

| DFT/B3LYP/6-311++G(2d,2p) )/(IEFPCM($CH_3CN$) | | |
|---|---|---|
| **Conformers** | **ΔG (Kcal/mol)** | **% Pop** |
| **C1** | 0.000000 | 41.4 |
| **C2** | 0.299079 | 25.0 |
| **C3** | 0.493449 | 18.0 |
| **C4** | 0.581229 | 15.5 |

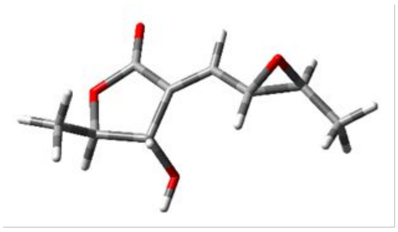

**C1**

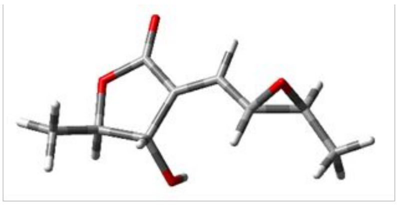

**C2**

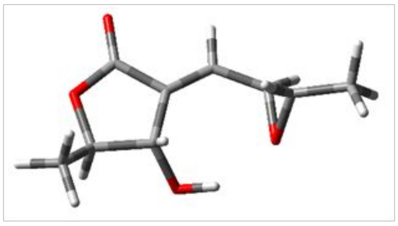

**C3**

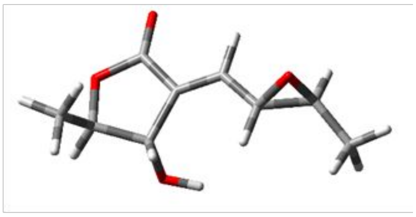

**C4**

**Figure S2.** Minimum energy conformers of (4*S*,5*R*,7*S*,8*S*)-**1b** computed at DFT/B3LYP/6-311++G(2d,2p)/IEFPCM($CH_3CN$) level. Conformers C3 displays intramolecular H-bond.

**Table S4.** Conformers Boltzmann distribution of (4*S*,5*R*,7*R*,8*S*)-**2a**.

| DFT/B3LYP/6-311++G(2d,2p) )/(IEFPCM($CH_3CN$) | | |
|---|---|---|
| **Conformers** | **ΔG (Kcal/mol)** | **% Pop** |
| **C1** | 0.000000 | 59.9 |
| **C2** | 0.795663 | 15.6 |
| **C3** | 0.800679 | 15.5 |
| **C4** | 1.131108 | 8.9 |

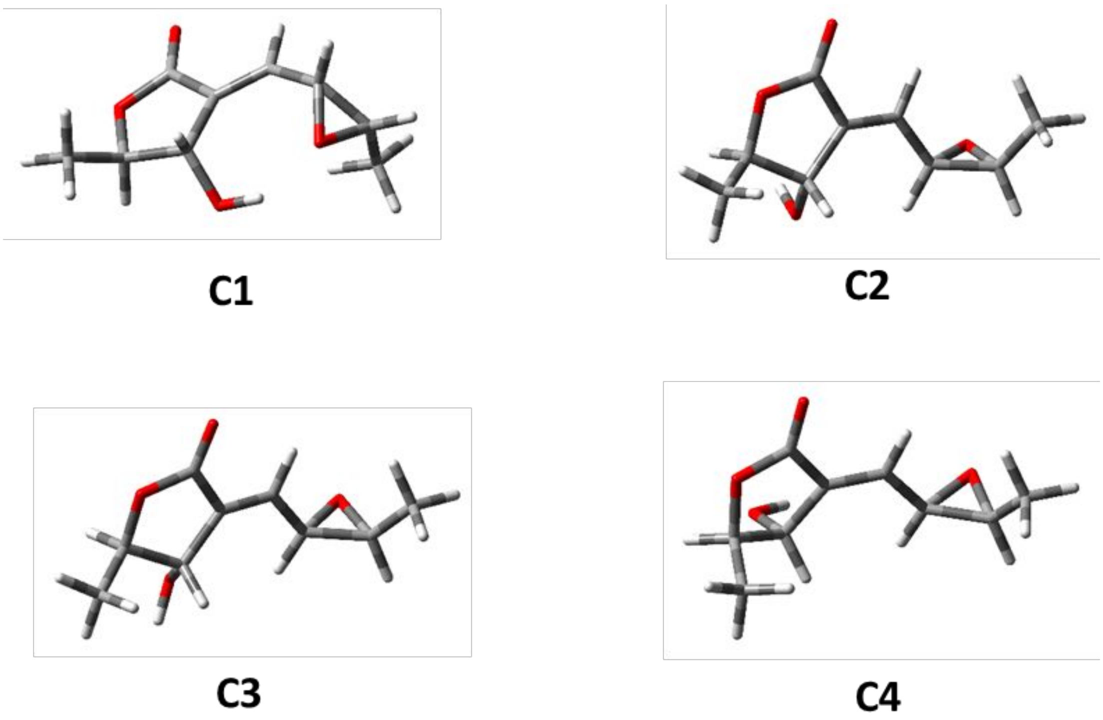


**Figure S3.** Structures of conformers found of (4*S*,5*R*,7*R*,8*S*)-**2a** computed at DFT/B3LYP/6-311++G(2d,2p)/IEFPCM($CH_3CN$) level. Conformers C1 displays intramolecular H-bond.

**Table S5.** Conformers Boltzmann distribution of (4*S*,5*R*,7*S*,8*R*)-**2b**.

| DFT/B3LYP/6-311++G(2d,2p) )/(IEFPCM($CH_3CN$) | | |
|---|---|---|
| **Conformers** | **ΔG (Kcal/mol)** | **% Pop** |
| **C1** | 0.000000 | 35.8 |
| **C2** | 0.282777 | 22.2 |
| **C3** | 0.426360 | 17.4 |
| **C4** | 0.537339 | 14.4 |
| **C5** | 0.741741 | 10.2 |

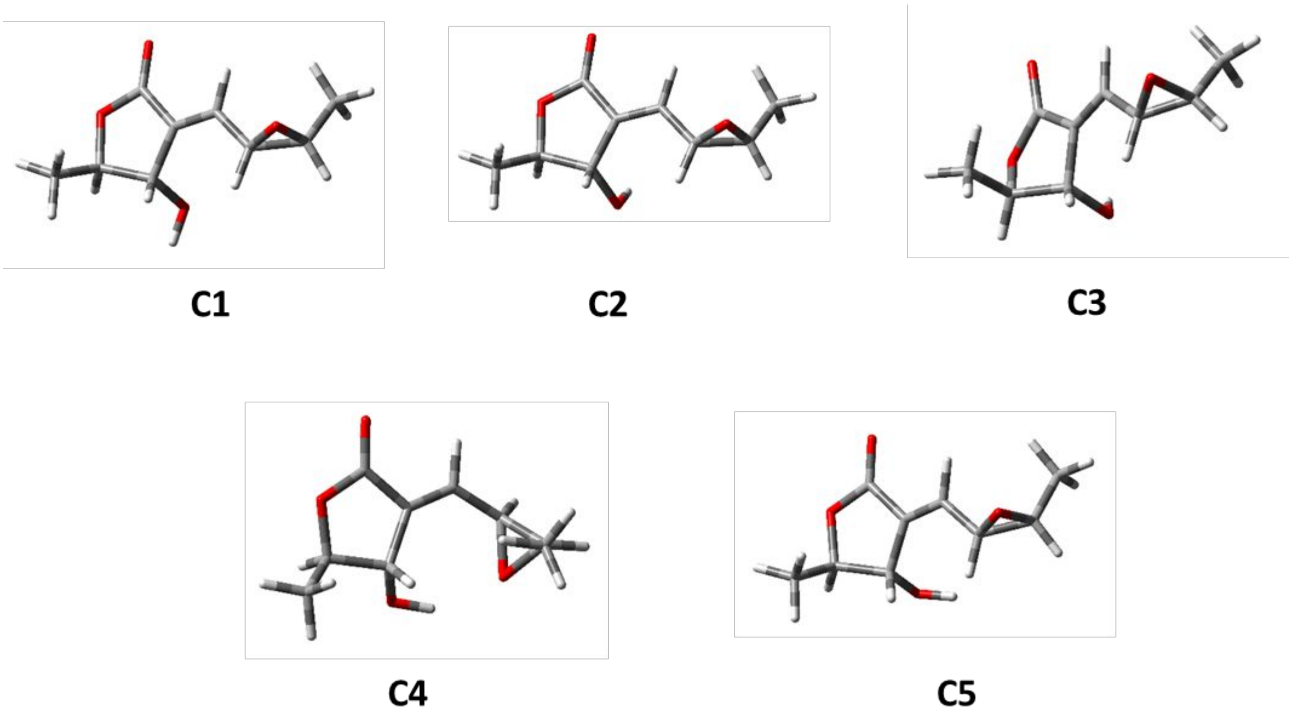


**Figure S4.** Structures of conformers found of (4*S*,5*R*,7*S*,8*R*)-**2b** computed at DFT/B3LYP/6-311++G(2d,2p)/IEFPCM($CH_3CN$) level. Conformers C4 displays intramolecular H-bond.

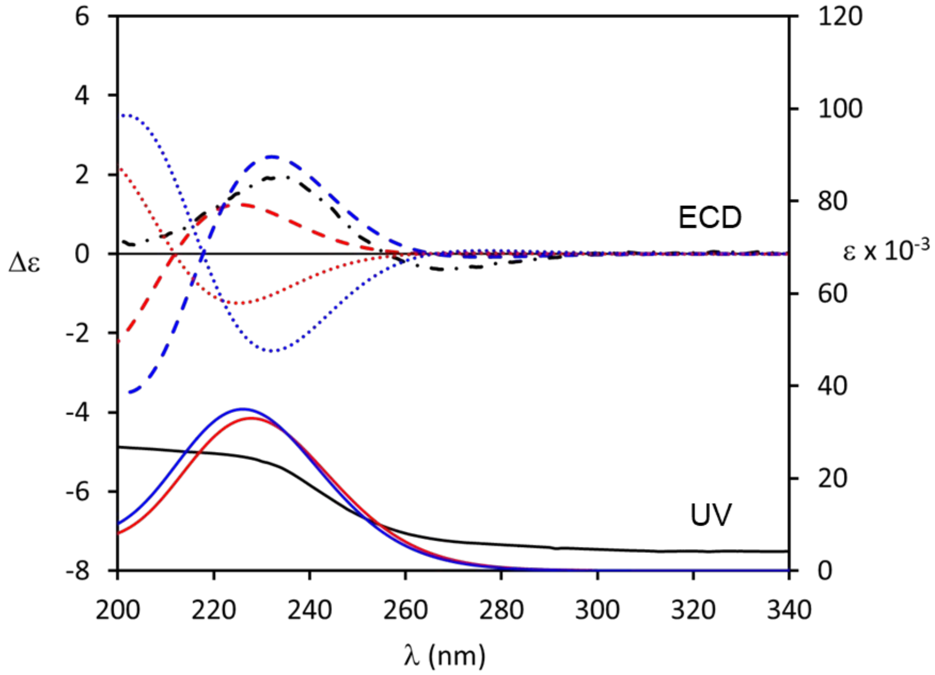


**Figure S5.** Comparison between experimental UV (solid black lines) and ECD spectra (dashed-dotted black line) of (+)-**1** with calculated [TDDFT/CAM-B3LYP/aug-cc-pVDZ/IEFPCM($CH_3CN$)] ones. Computed UV spectrum for **1a** (solid red line) and **1b** (solid blue line). Computed ECD spectrum for (4*S*,5*R*,7*R*,8*R*)-**1a** (dotted red line), (4*R*,5*S*,7*S*,8*S*)-**ent-1a** (dashed red line), (4*S*,5*R*,7*S*,8*S*)-**1b** (dotted blue line), and (4*R*,5*S*,7*R*,8*R*)-**ent-1b** (dashed blue line). The calculated ECD spectra were divided by 2.

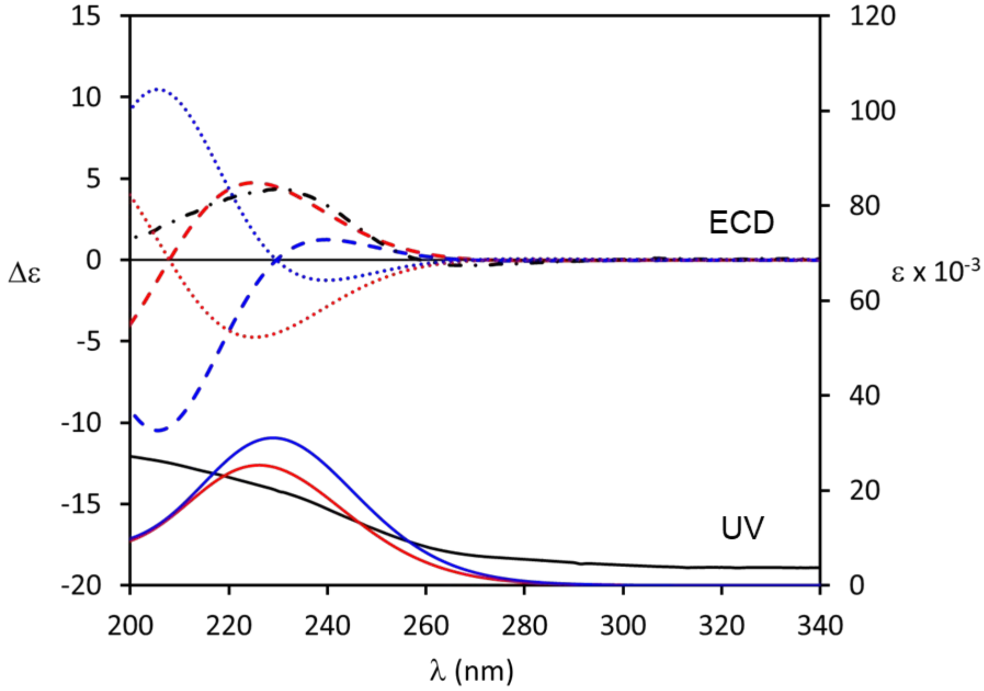


**Figure S6.** Comparison between experimental UV (solid black lines) and ECD (dashed-dotted black line) spectra of (+)-**2** with calculated [TDDFT/CAM-B3LYP/aug-cc-pVDZ/IEFPCM($CH_3CN$)] ones. Computed UV spectrum for **2a** (solid red line) and **2b** (solid blue line). Computed ECD spectrum for (4*S*,5*R*,7*R*,8*S*)-**2a** (dotted red line), (4*R*,5*S*,7*S*,8*R*)-**ent-2a** (dashed red line), (4*S*,5*R*,7*S*,8*R*)-**2b** (dotted blue line), and (4*R*,5*S*,7*R*,8*S*)-**ent-2b** (dashed blue line).

*ECD computations with explicit solvent model.*

To further confirm the absolute configuration assignment the ECD spectra were also carried out ECD computations by simulating the solvent effects in an explicit approach. In fact, it is known that even with the weak hydrogen bond accepting solvent acetonitrile a better description of the solvent-solute interaction is often obtained by performing computations in the explicit solvent mode, i.e. including one or more solvent molecules in the input structures. Computational conformational analysis was then repeated at DFT/B3LYP/6-311++G(2d,2p) level of theory, adding a single molecule of acetonitrile H-bonded to the OH moiety of (4*S*,5*R*,7*R*,8*R*)**-1a** and (4*S*,5*R*,7*S*,8*S*)**-1b**. Four populated conformers were obtained for both diastereomers (see Table S5, Figure S7, Table S6, and Figure S8). Those displaying intramolecular H-bonding were again discarded and UV and ECD spectra were computed at TDDFT/CAM-B3LYP/aug-cc-pVDZ level on the remaining structures. Comparison of the UV and ECD spectra computed in the explicit solvent mode with the experimental spectra (Figure S9) supports the above assignment of (4*R*,5*S*,7*R*,8*R*) AC to pinofuranoxin A (+)-(**1**). The same analysis, employing the explicit solvent approach, has been performed also for pinofuranoxin B (**2**). For this compound four populated conformers were obtained for both diastereomers (4*S*,5*R*,7*R*,8*S*)-**2a** and (4*S*,5*R*,7*S*,8*R*)-**2b** (see Table S7, Figure S10, Table S8, and Figure S11). Those displaying intramolecular H-bonding were again discarded and UV and ECD spectra were computed on the remaining structures. Comparison of the UV and ECD spectra computed in the explicit solvent mode with the experimental spectra (Figure S12) supports the above assignment of (4*R*,5*S*,7*S*,8*R*) AC to pinofuranoxin B (+)-(**2**).

**Table S6.** Conformers Boltzmann distribution of (4*S*,5*R*,7*R*,8*R*)-**1a·ACN** adduct.

| DFT/B3LYP/6-311++G(2d,2p) | | |
|---|---|---|
| **Conformers** | **ΔG (Kcal/mol)** | **% Pop** |
| **C1** | 0.000000 | 67.0 |
| **C2** | 0.674652 | 21.5 |
| **C3** | 1.302279 | 7.4 |
| **C4** | 1.654026 | 4.1 |

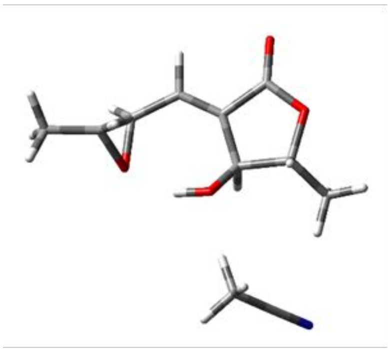

**C1**

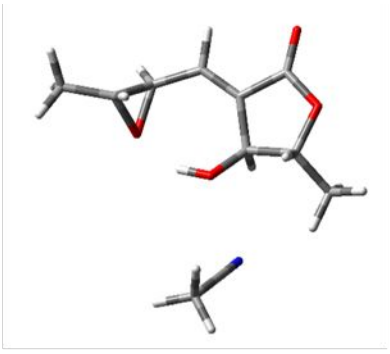

**C2**

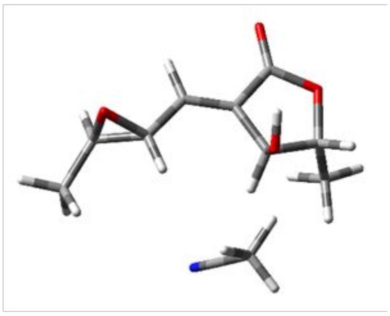

**C3**

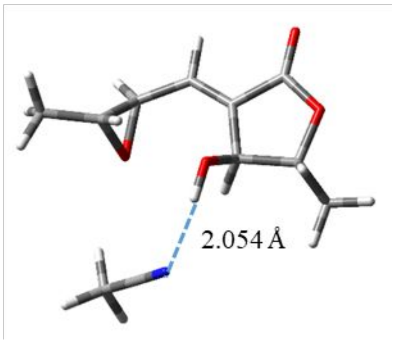


**C4**

**Figure S7.** Structures of most stable conformers of (4*S*,5*R*,7*R*,8*R*)-**1a·ACN** adduct calculated at DFT/B3LYP/6-311++G(2d,2p) level of theory. Conformers C1 and C2 display intramolecular H-bond, while conformer C4 intermolecular H-bond with acetonitrile.

**Table S7.** Conformers Boltzmann distribution of (4*S*,5*R*,7*S*,8*S*)-**1b·ACN** adduct.

| | DFT/B3LYP/6-311++G(2d,2p) | |
|---|---|---|
| **Conformers** | **ΔG (Kcal/mol)** | **% Pop** |
| **C1** | 0.000000 | 78.0 |
| **C2** | 1.183149 | 10.6 |
| **C3** | 1.350558 | 8.0 |
| **C4** | 1.854039 | 3.4 |

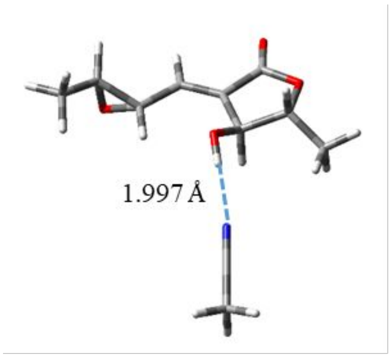


**C1**

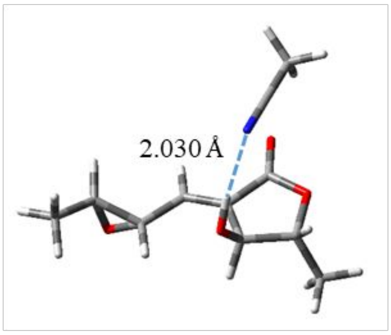


**C2**

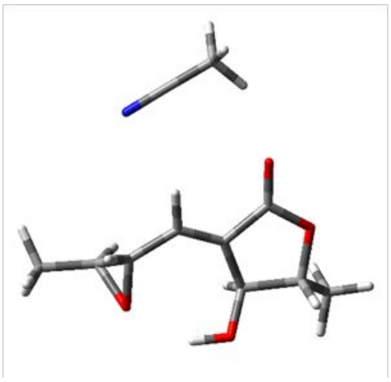

**C3**

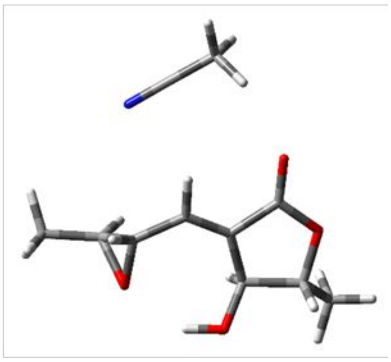

**C4**

**Figure S8.** Structures of most stable conformers of (4*S*,5*R*,7*S*,8*S*)-**1b·ACN** adduct calculated at DFT/B3LYP/6-311++G(2d,2p) level of theory. Conformers C3 and C4 display intramolecular H-bond, while conformers C1 and C2 intermolecular H-bond with acetonitrile.

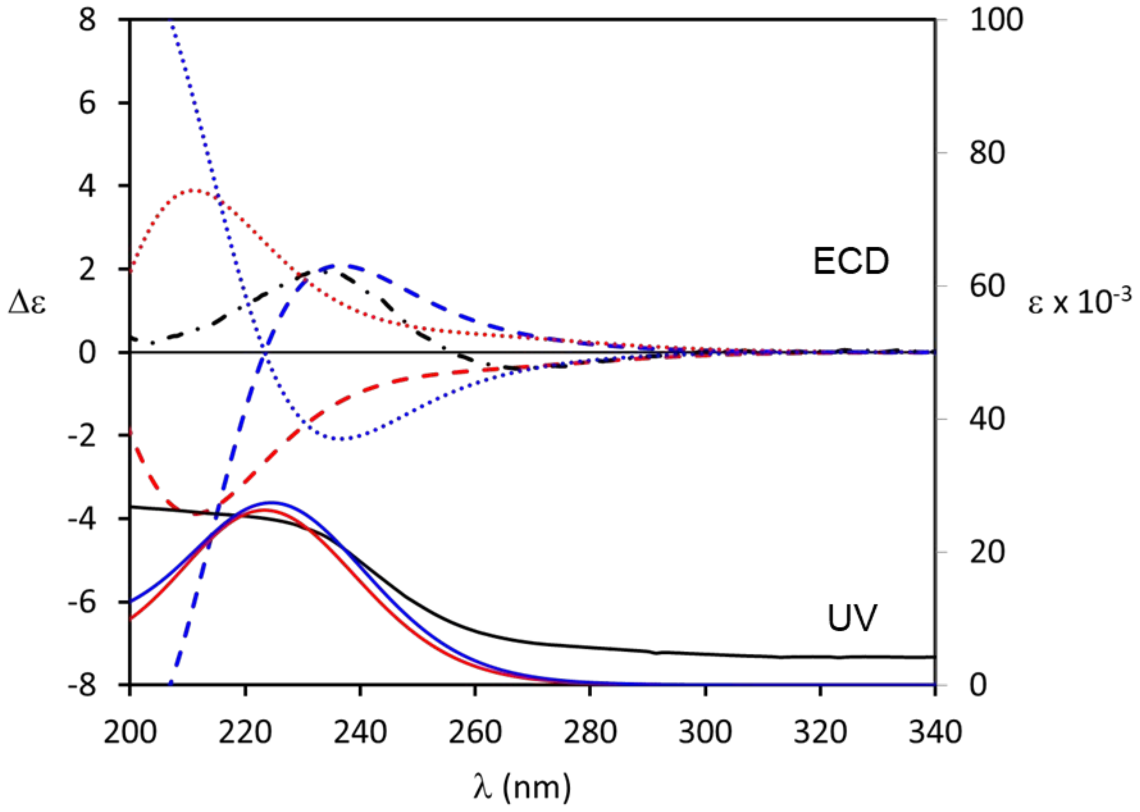


**Figure S9.** Comparison between experimental UV (solid black lines) and ECD (dashed-dotted black line) spectra of (+)-**1** with calculated [TDDFT/CAM-B3LYP/aug-cc-pVDZ/explicit model($CH_3CN$)] ones. Computed UV spectrum for **1a·ACN** adduct (solid red line) and **1b·ACN** adduct (solid blue line). Computed ECD spectrum for (4*S*,5*R*,7*R*,8*R*)-**1a·ACN** adduct (dotted red line), (4*R*,5*S*,7*S*,8*S*)-**ent-1a·ACN** adduct (dashed red line), (4*S*,5*R*,7*S*,8*S*)-**1b·ACN** adduct (dotted blue line), and (4*R*,5*S*,7*R*,8*R*)-**ent-1b·ACN** adduct (dashed blue line). The calculated ECD spectra were divided by 2. Conformers with intramolecular hydrogen bonding have been removed (see text).

**Table S8.** Conformers Boltzmann distribution of (4*S*,5*R*,7*R*,8*S*)-**2a·ACN** adduct.

| | DFT/B3LYP/6-311++G(2d,2p) | |
|---|---|---|
| **Conformers** | **ΔG (Kcal/mol)** | **% Pop** |
| **C1** | 0.000000 | 49.4 |
| **C2** | 0.231363 | 33.4 |
| **C3** | 0.675279 | 15.8 |
| **C4** | 2.109855 | 1.4 |

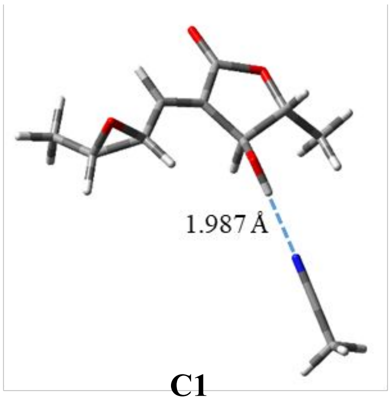


**C1**

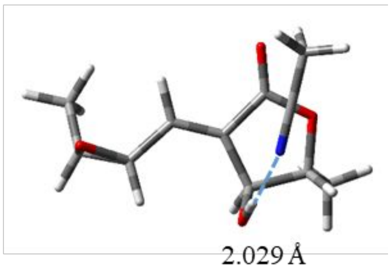


**C2**

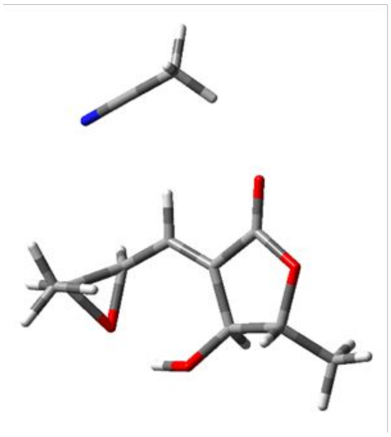

**C3**

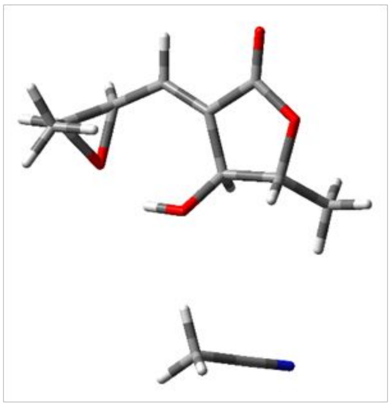

**C4**

**Figure S10.** Structures of most stable conformers of (4*S*,5*R*,7*R*,8*S*)-**2a·ACN** adduct calculated at DFT/B3LYP/6-311++G(2d,2p) level of theory. Conformers C3 and C4 display intramolecular H-bond, while conformers C1 and C2 intermolecular H-bond with acetonitrile.

**Table S9.** Conformers Boltzmann distribution of (4*S*,5*R*,7*S*,8*R*)-**2b·ACN** adduct.

| | DFT/B3LYP/6-311++G(2d,2p) | |
|---|---|---|
| **Conformers** | **ΔG (Kcal/mol)** | **% Pop** |
| **C1** | 0.000000 | 55.8 |
| **C2** | 0.158631 | 42.7 |
| **C3** | 2.576970 | 0.7 |
| **C4** | 2.594526 | 0.7 |

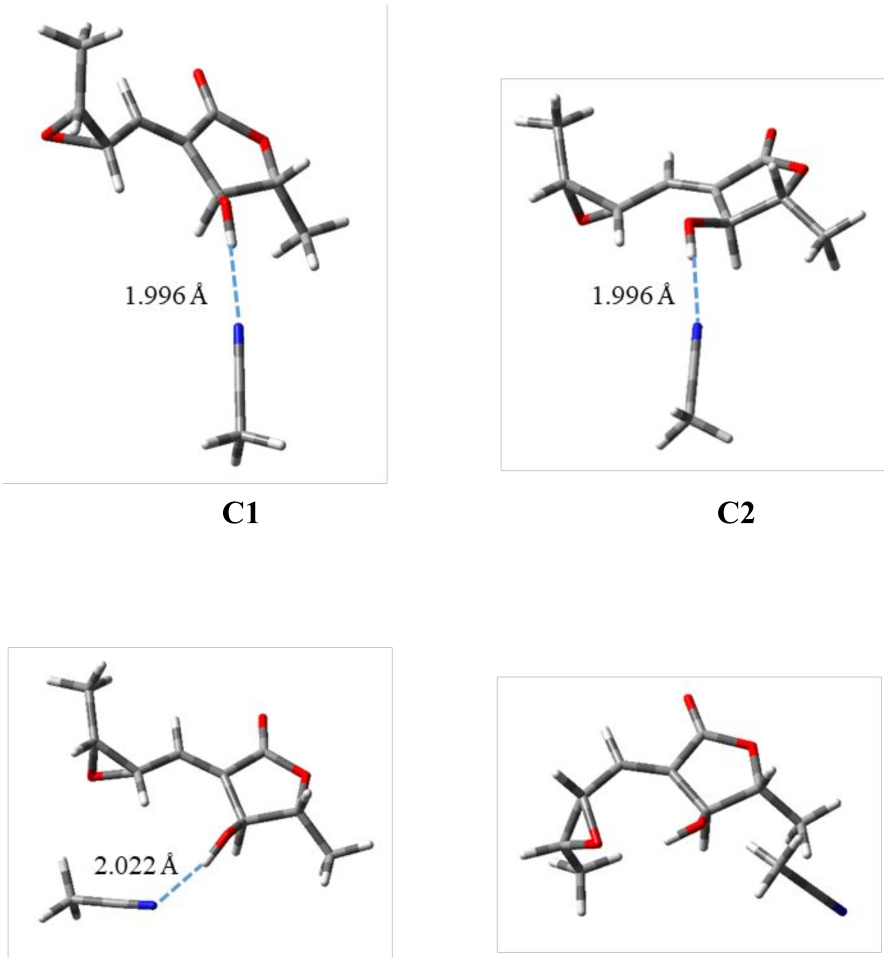


**C1** **C2**

**C3** **C4**

**Figure S11.** Structures of most stable conformers (4*S*,5*R*,7*S*,8*R*)-**2b·ACN** adduct calculated at DFT/B3LYP/6-311++G(2d,2p) level of theory. Conformers displays intramolecular H-bond, while conformers C1, C2, and C3 intermolecular H-bond with acetonitrile.

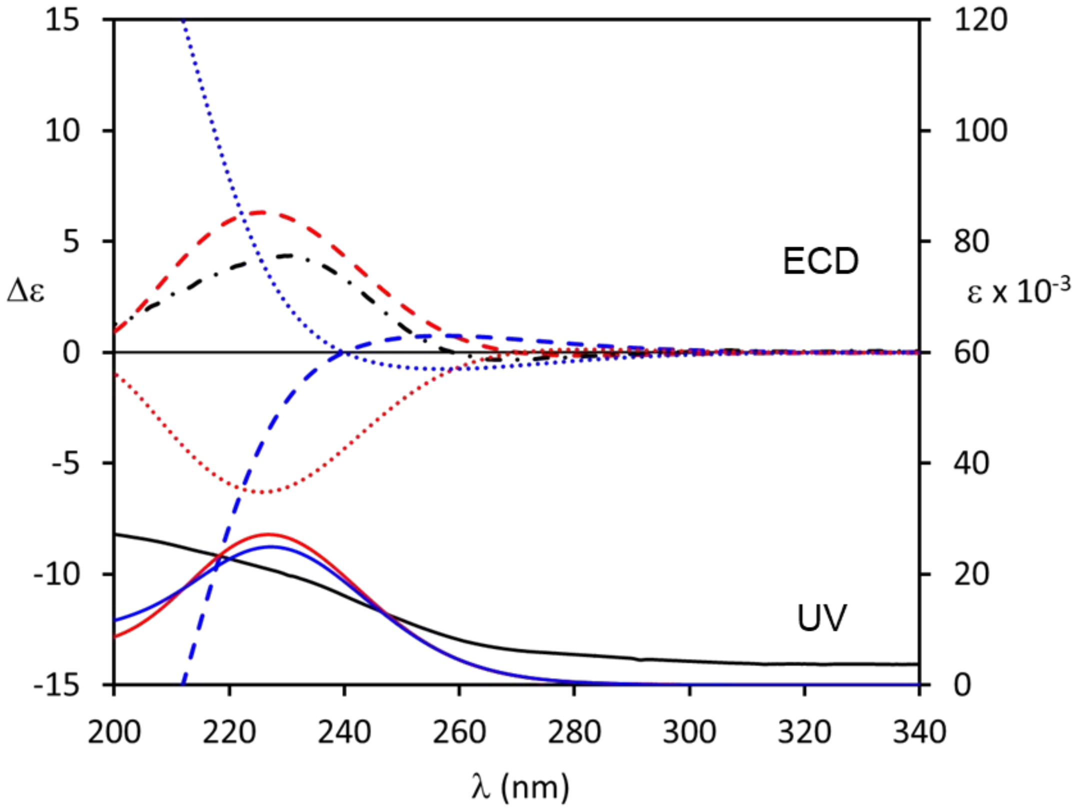


**Figure S12.** Comparison between experimental UV (solid black lines) and ECD (dashed-dotted black line) spectra of (+)-**2** with calculated [TDDFT/CAM-B3LYP/aug-cc-pVDZ/explicit model($CH_3CN$)] ones. Computed UV spectrum for **2a·ACN** adduct (solid red line) and **2b·ACN** adduct (solid blue line). Computed ECD spectrum for (4*S*,5*R*,7*R*,8*S*)-**2a·ACN** adduct (dotted red line), (4*R*,5*S*,7*S*,8*R*)-**ent-2a·ACN** adduct (dashed red line), (4*S*,5*R*,7*S*,8*R*)-**2b·ACN** adduct (dotted blue line), and (4*R*,5*S*,7*R*,8*S*)-**ent-2b·ACN** adduct (dashed blue line). Conformers with intramolecular hydrogen bonding have been removed (see text).

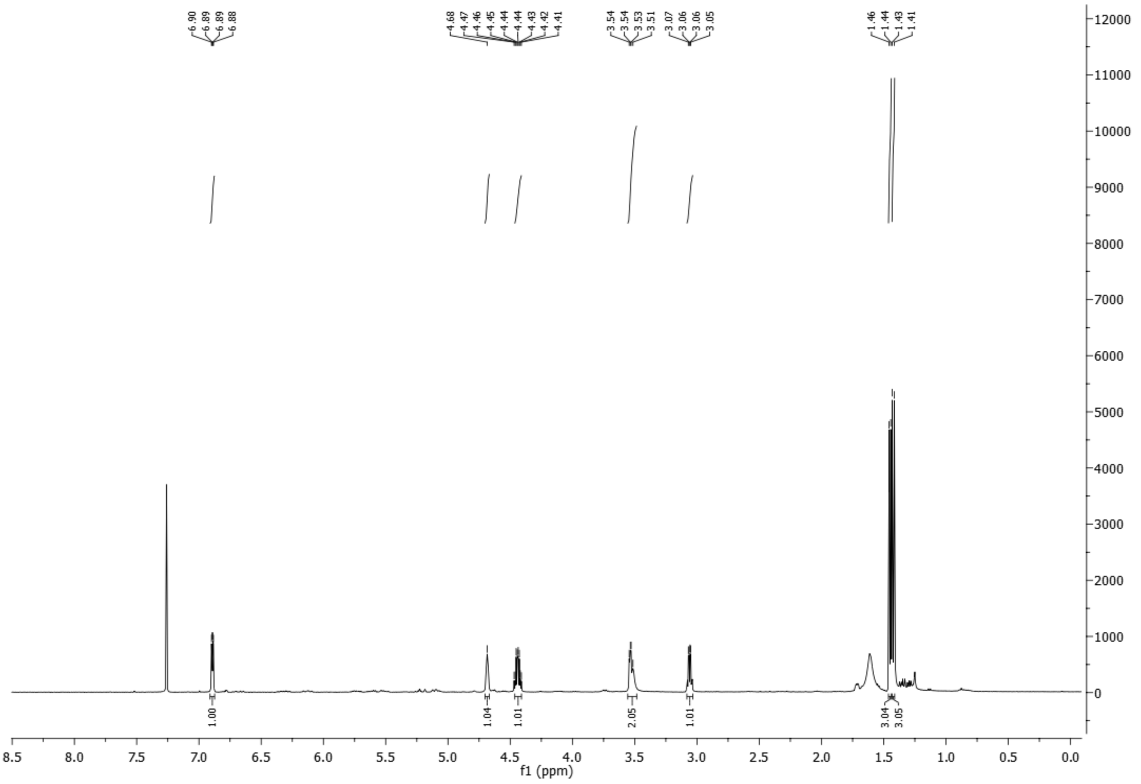


**Spectrum 1.** $^{1}$H NMR spectrum of pinofuranoxin A (**1**) ($CDCl_3$, 400 MHz).

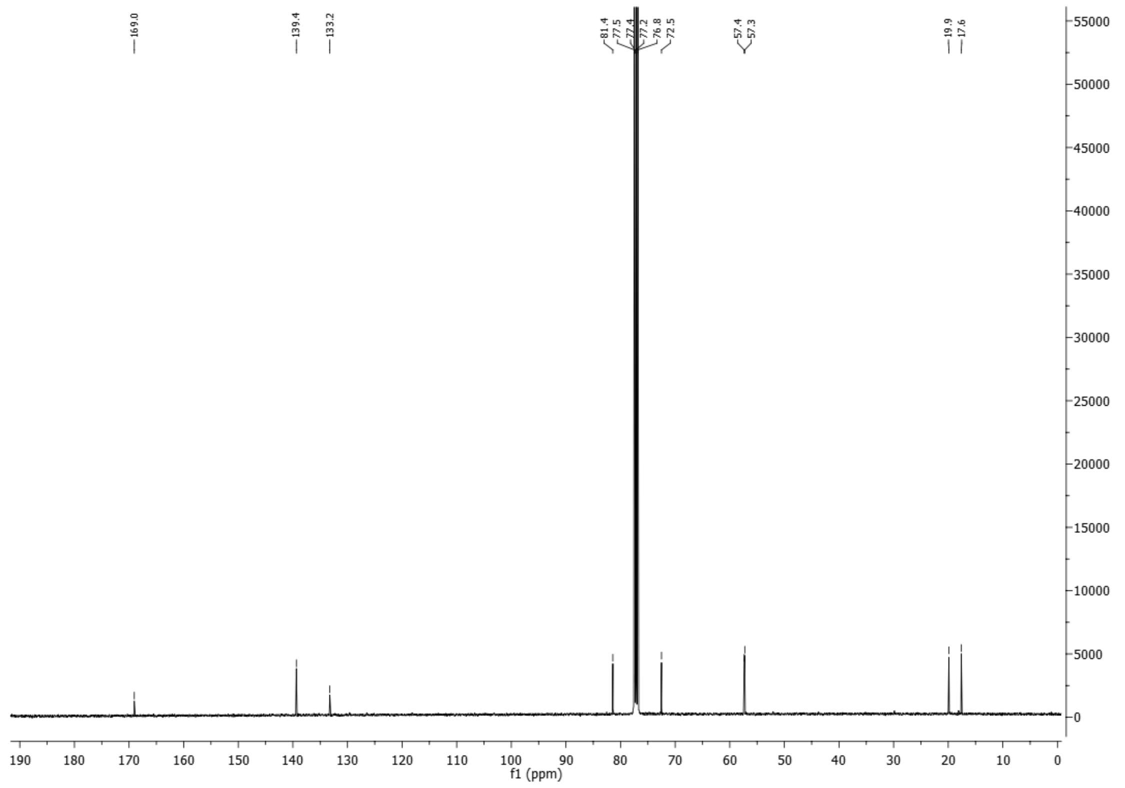


**Spectrum 2.** $^{13}$C NMR spectrum of pinofuranoxin A (**1**) ($CDCl_3$, 100 MHz).

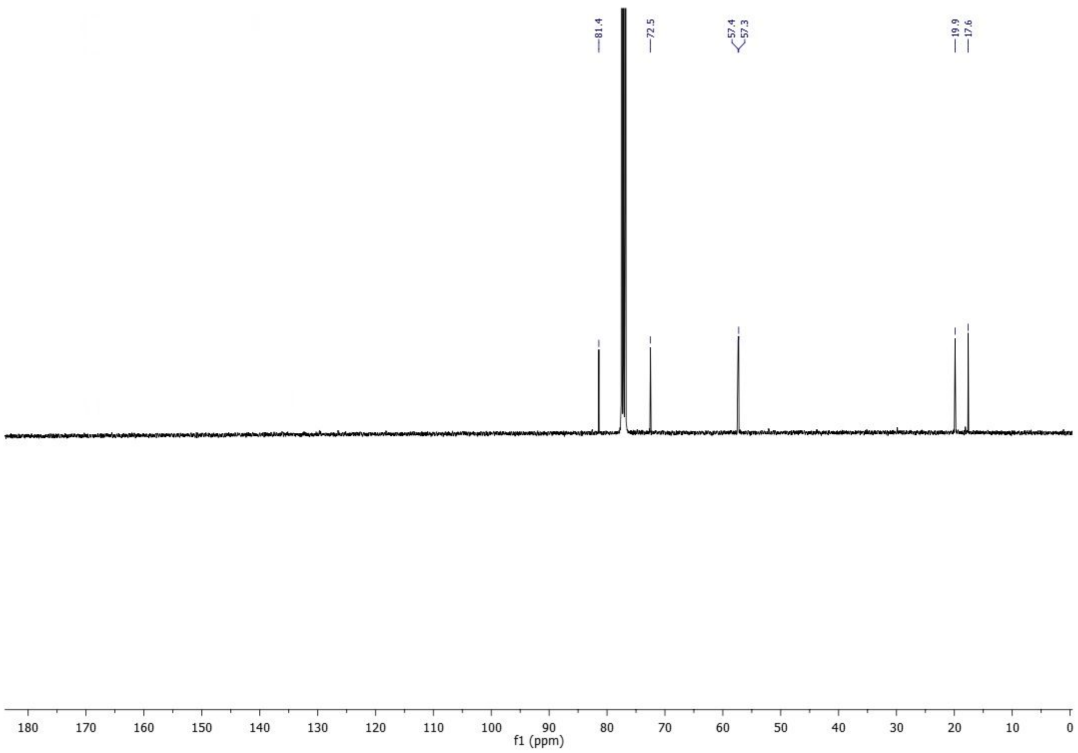


**Spectrum 3.** DEPT-135 NMR spectrum of pinofuranoxin A (**1**) ($CDCl_3$, 100 MHz).

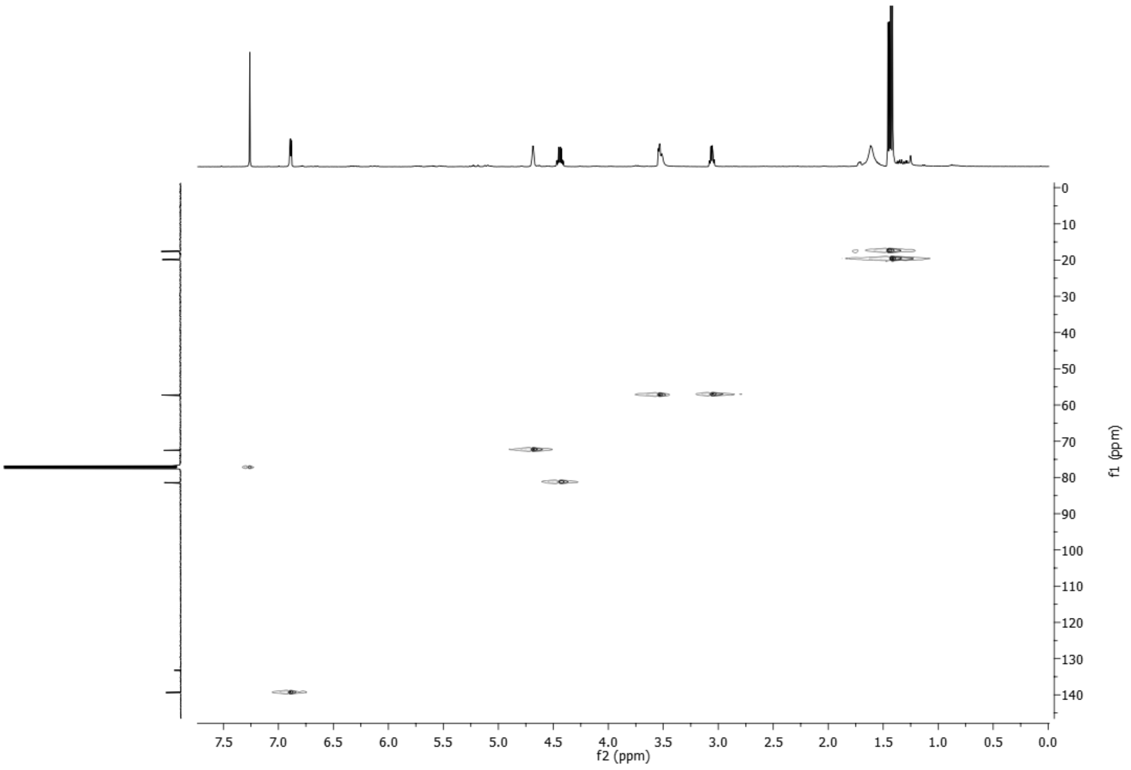


**Spectrum 4.** HSQC spectrum of pinofuranoxin A (**1**) ($CDCl_3$, 400/100 MHz).

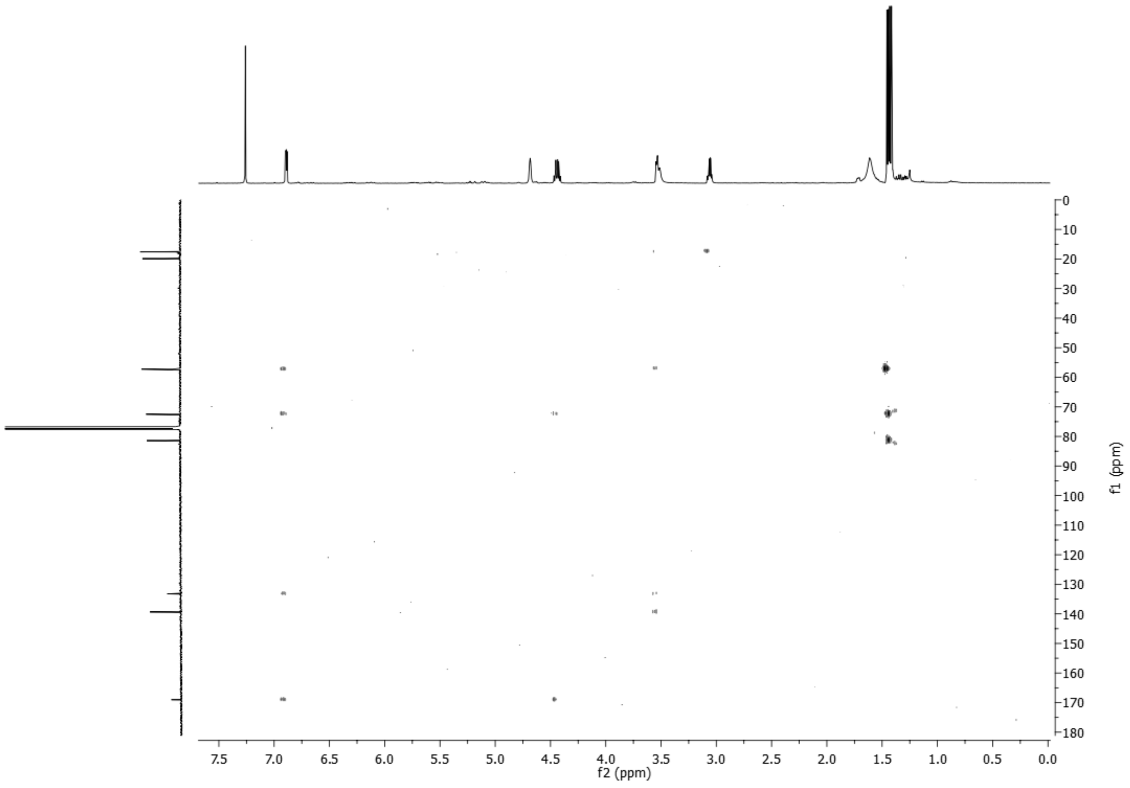


**Spectrum 5.** HMBC spectrum of pinofuranoxin A (**1**) ($CDCl_3$, 400/100 MHz).

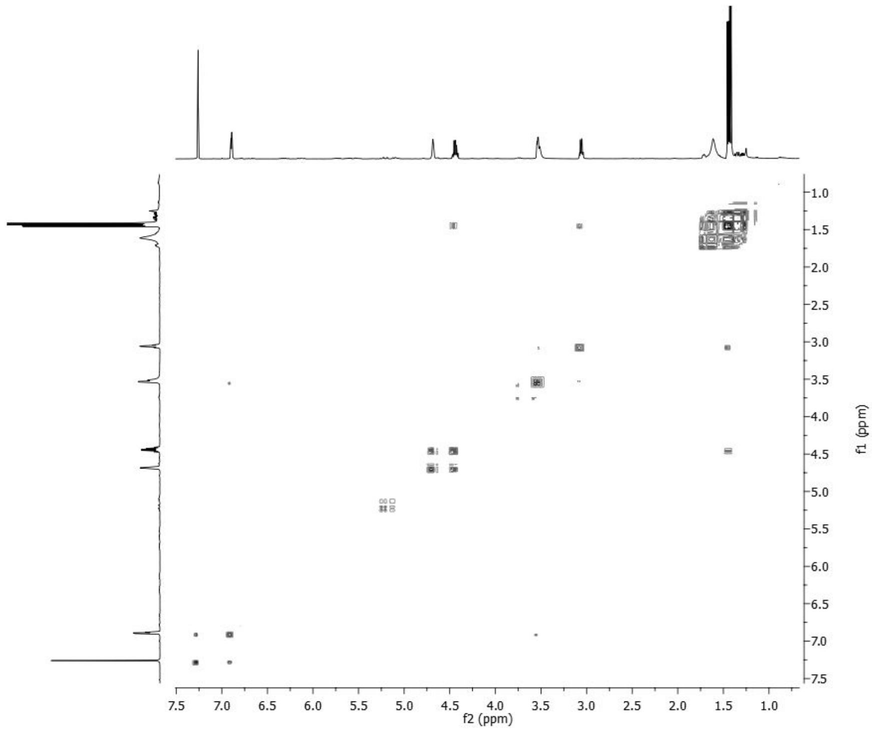


**Spectrum 6.** COSY spectrum of pinofuranoxin A (**1**) ($CDCl_3$, 400 MHz).

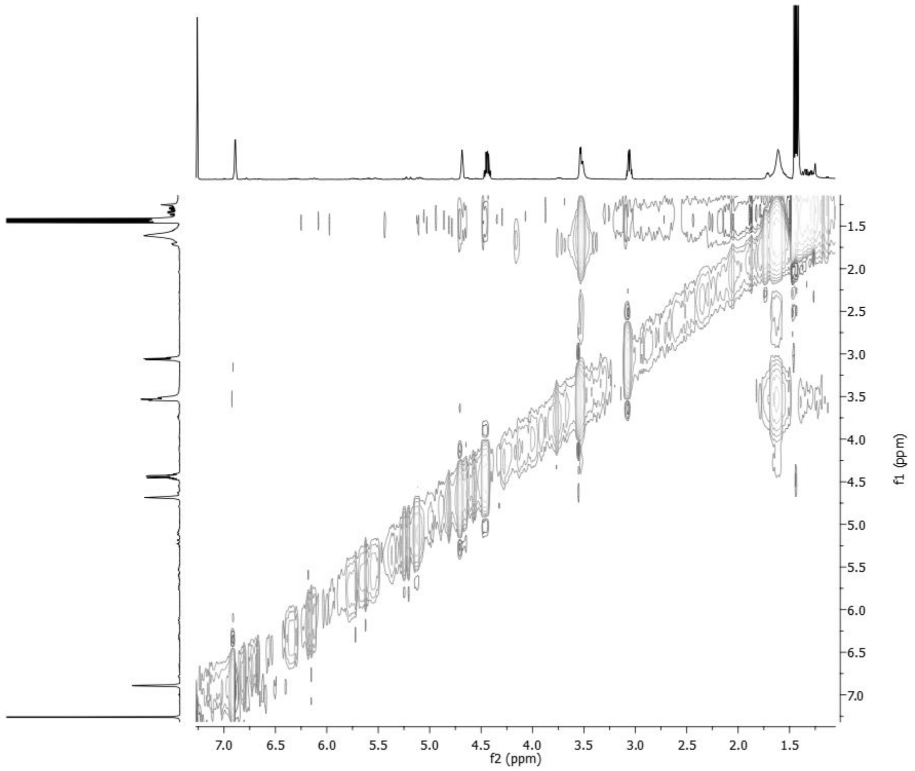


**Spectrum 7**. NOESY spectrum of pinofuranoxin A (**1**) ($CDCl_3$, 400 MHz).

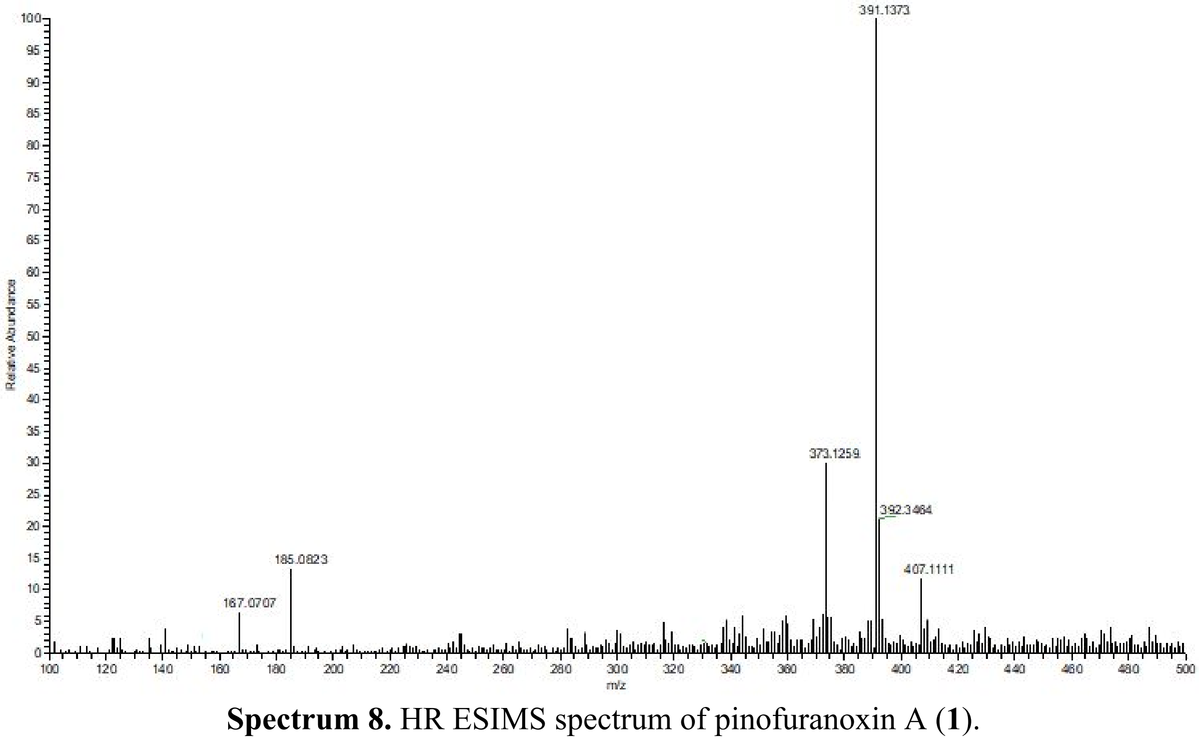


**Spectrum 8.** HR ESIMS spectrum of pinofuranoxin A (**1**).

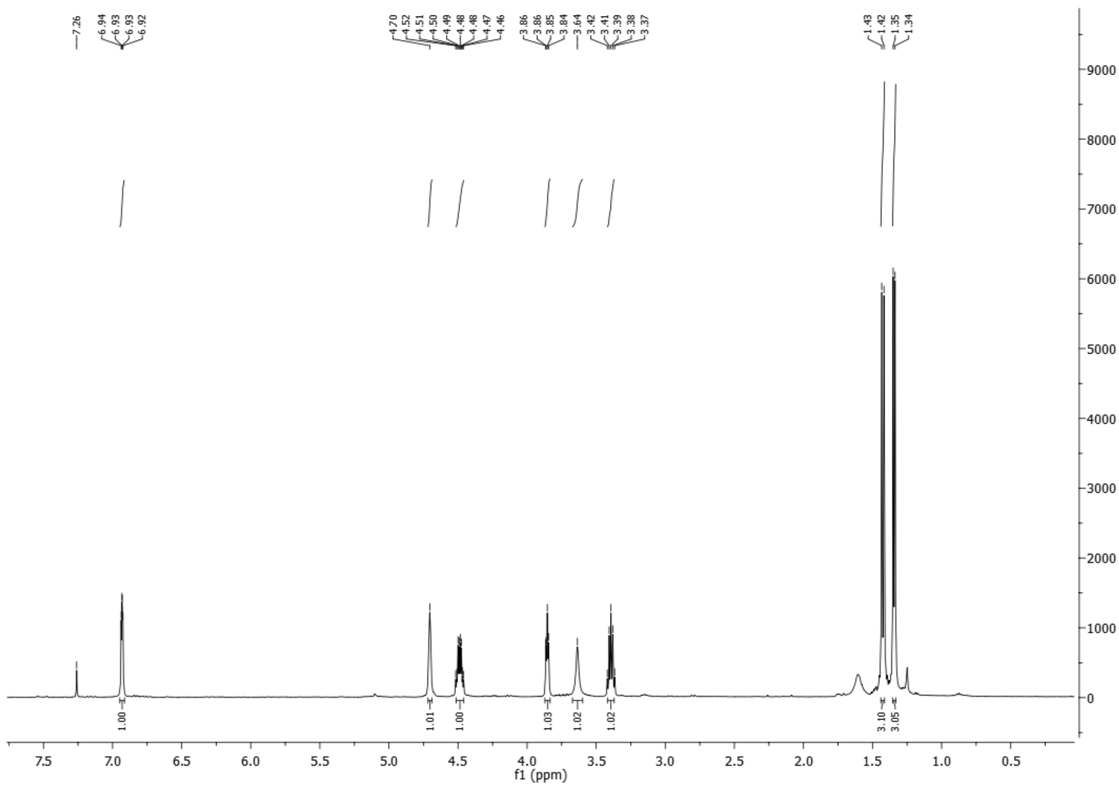

**Spectrum 9**. $^{1}H$ NMR spectrum of pinofuranoxin B (**2**) ($CDCl_3$, 400 MHz).

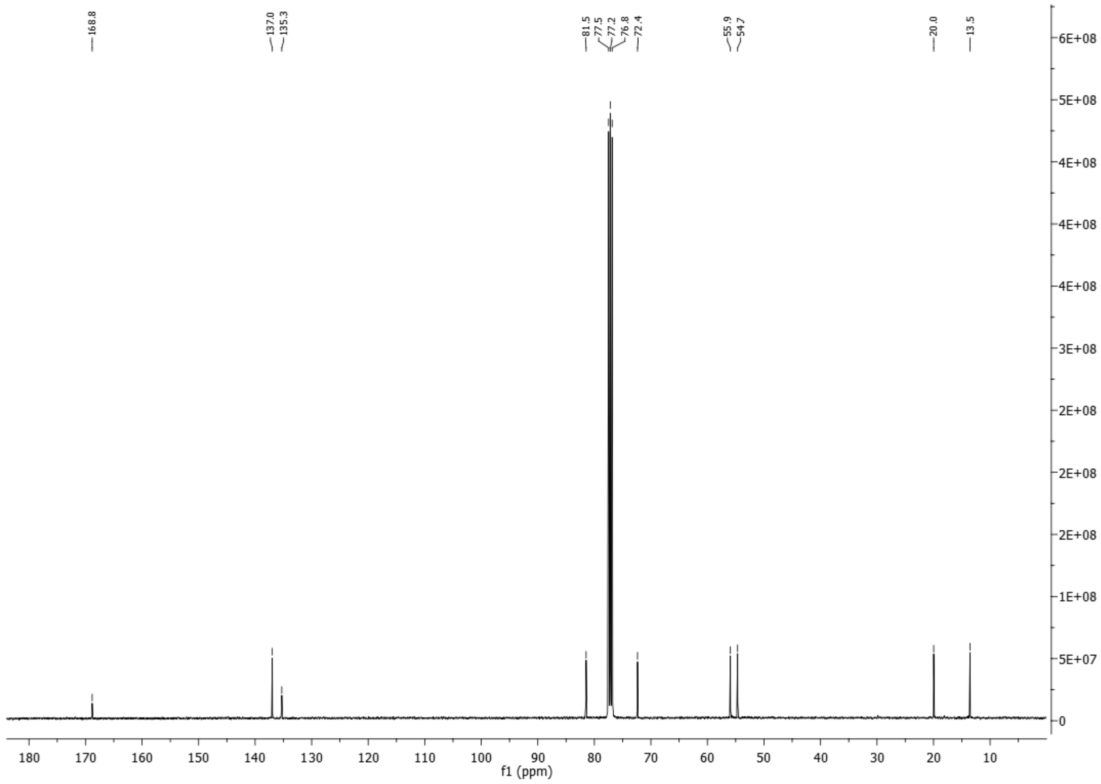

**Spectrum 10**. $^{13}C$ NMR spectrum of pinofuranoxin B (**2**) ($CDCl_3$, 100 MHz).

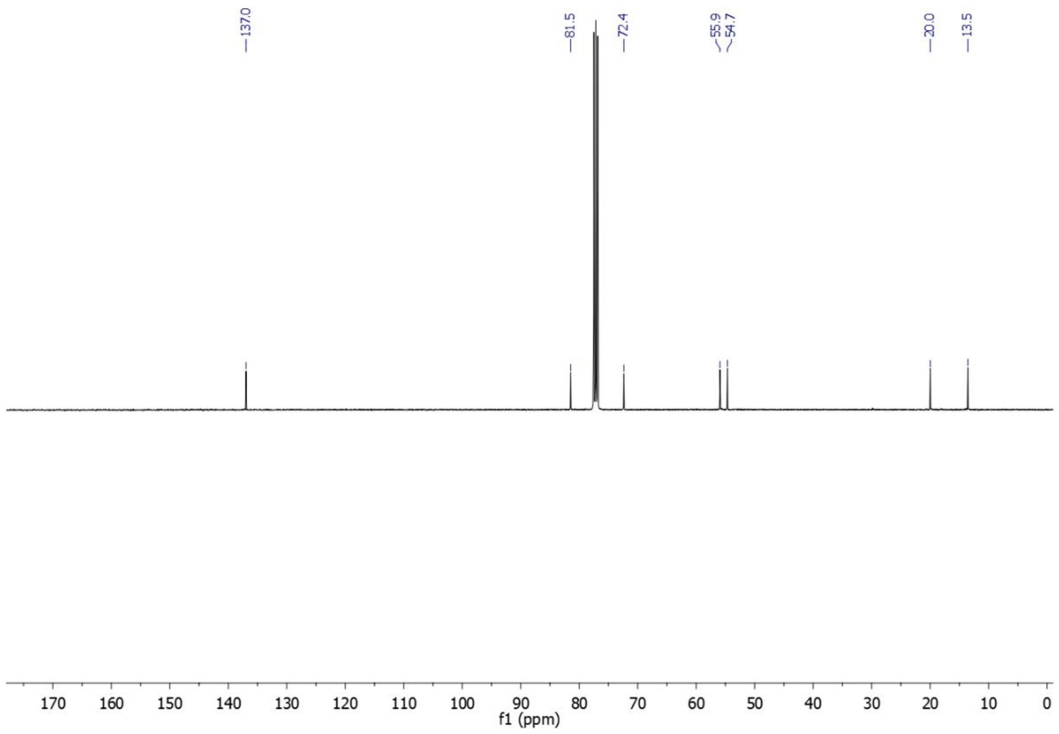


**Spectrum 11.** DEPT-135 NMR spectrum of pinofuranoxin B (**2**) ($CDCl_3$, 100 MHz).

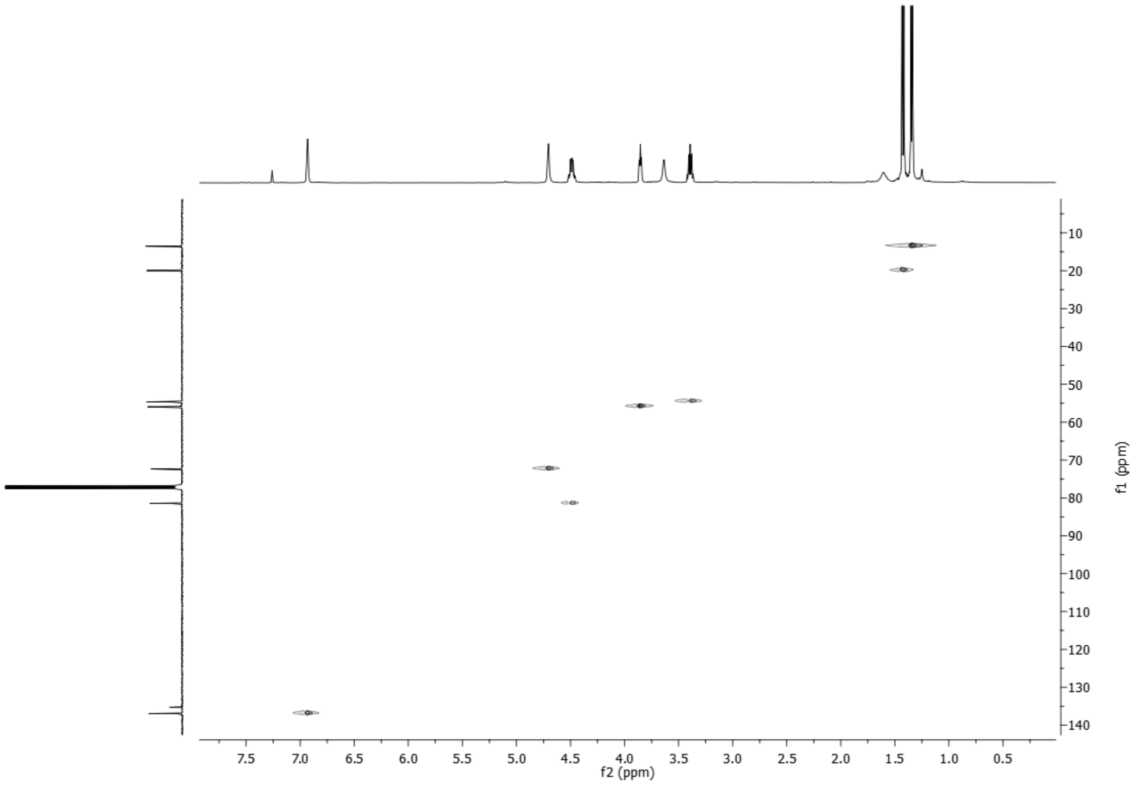


**Spectrum 11**. HSQC spectrum of pinofuranoxin B (**2**) ($CDCl_3$, 400/100 MHz).

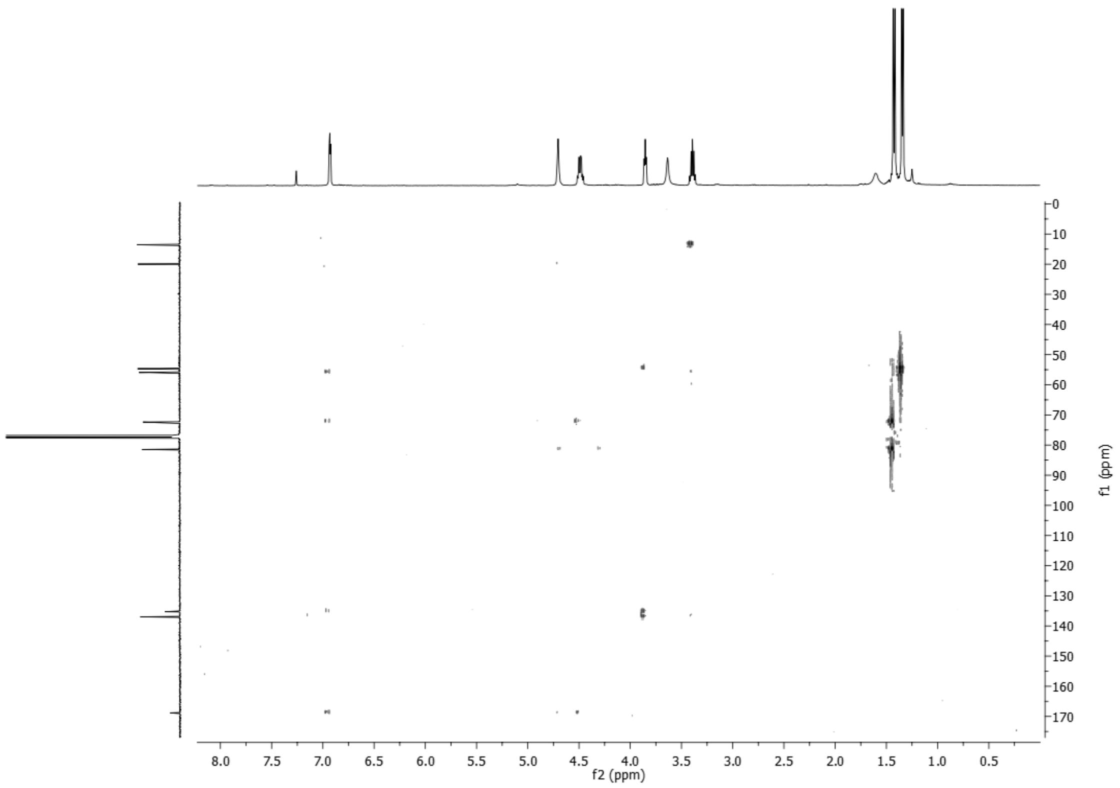


**Spectrum 11**. HMBC spectrum of pinofuranoxin B (**2**) ($CDCl_3$, 400/100 MHz).

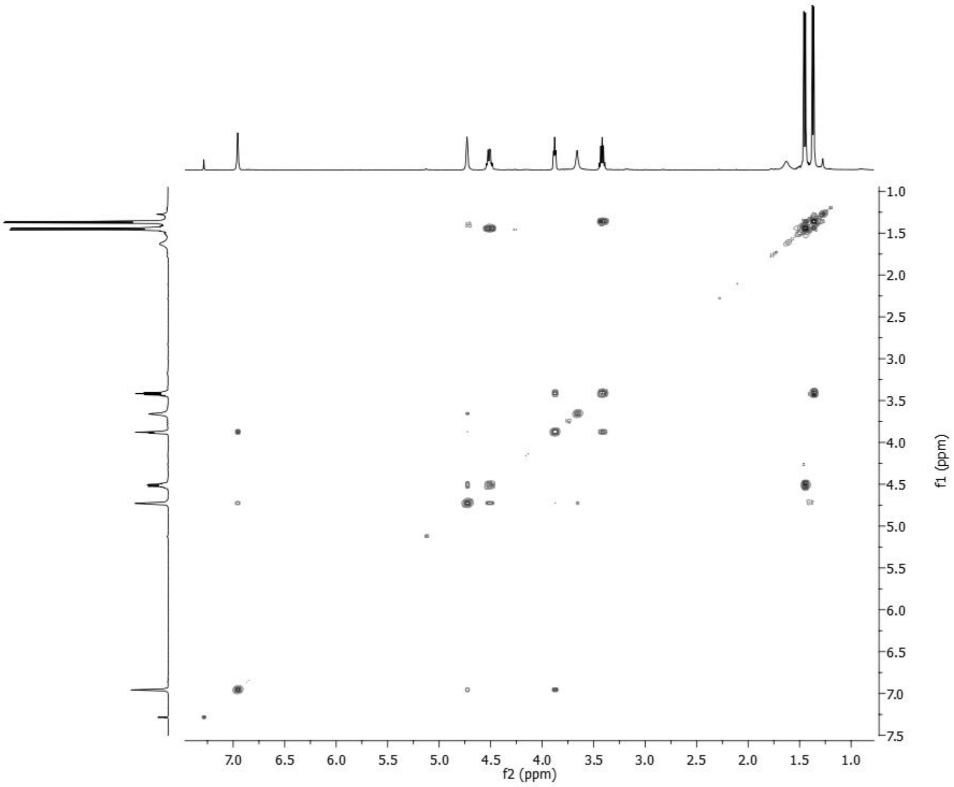


**Spectrum 12**. COSY spectrum of pinofuranoxin B (**2**) ($CDCl_3$, 400 MHz).

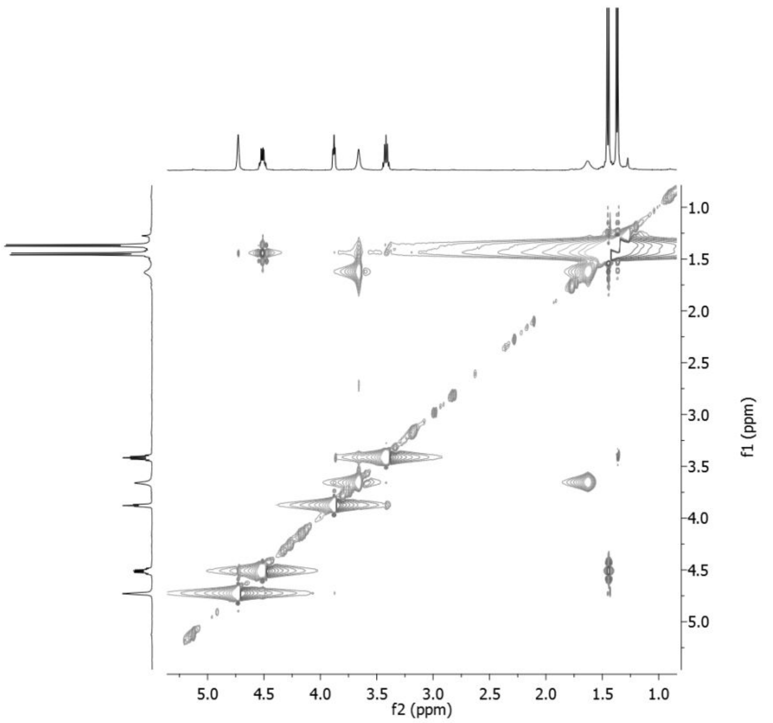


**Spectrum 13**. NOESY spectrum of pinofuranoxin B (**2**) ($CDCl_3$, 400 MHz).

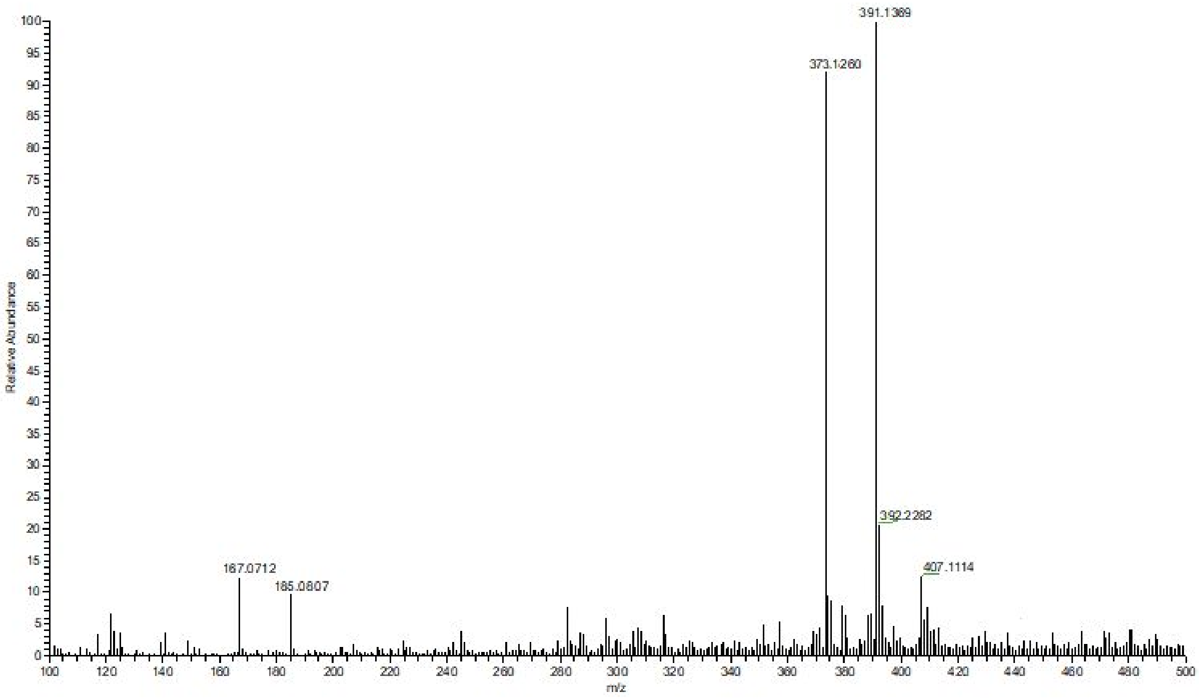


**Spectrum 14.** HR ESIMS spectrum of pinofuranoxin B (**2**).
